# Identification of LINC02454-related key pathways and genes in papillary thyroid cancer by weighted gene coexpression network analysis (WGCNA)

**DOI:** 10.1186/s13044-024-00205-8

**Published:** 2024-09-02

**Authors:** Yingjian Song, Lin Wang, Yi Ren, Xilei Zhou, Juan Tan

**Affiliations:** 1 Department of Respiratory and Critical Care Medicine, The Affiliated Huaian No. 1 People’s Hospital of Nanjing Medical University, Huaian, Jiangsu China; 2 Department of Breast and Thyroid Surgery, The Affiliated Huaian No. 1 People’s Hospital of Nanjing Medical University, Huaian, Jiangsu China; 3 Department of Radiation Oncology, The Affiliated Huaian No. 1 People’s Hospital of Nanjing Medical University, Huai’an, Jiangsu, China; 4 Department of General Practice, The Affiliated Huaian No. 1 People’s Hospital of Nanjing Medical University, 6 Beijing Road West, Huaian, 223300 Jiangsu China

**Keywords:** Long non-coding RNA, Papillary thyroid cancer, Key pathway, Key genes

## Abstract

**Background:**

Our previous study demonstrated that long intergenic noncoding RNA 02454 (LINC02454) may act as an oncogene to promote the proliferation and inhibit the apoptosis of papillary thyroid cancer (PTC) cells. This study was designed to investigate the mechanisms whereby LINC02454 is related to PTC tumorigenesis.

**Methods:**

Thyroid cancer RNA sequence data were obtained from The Cancer Genome Atlas (TCGA) database. Weighted gene coexpression network analysis (WGCNA) was applied to identify modules closely associated with PTC. Kyoto Encyclopedia of Genes and Genomes (KEGG) pathway enrichment analysis was used to identify the key pathways, and the maximal clique centrality (MCC) topological method was used to identify the hub genes. The Gene Expression Profiling Interactive Analysis (GEPIA) database was used to compare expression levels of key genes between PTC samples and normal samples and explore the prognostic value of key genes. The key genes were further validated with GEO dataset.

**Results:**

The top 5000 variable genes were investigated, followed by an analysis of 8 modules, and the turquoise module was the most positively correlated with the clinical stage of PTC. KEGG pathway analysis found the top two pathways of the ECM − receptor interaction and MAPK signaling pathway. In addition, five key genes (FN1, LAMB3, ITGA3, SDC4, and IL1RAP) were identified through the MCC algorithm and KEGG analysis. The expression levels of the five key genes were significantly upregulated in thyroid cancer in both TCGA and GEO datasets, and of these five genes, FN1 and ITGA3 were associated with poor disease-free prognosis.

**Conclusions:**

Our study identified five key genes and two key pathways associated with LINC02454, which might shed light on the underlying mechanism of LINC02454 action in PTC.

**Supplementary Information:**

The online version contains supplementary material available at 10.1186/s13044-024-00205-8.

## Introduction

Thyroid cancer is the most common endocrine cancer worldwide, with an estimated 44 020 cases and 2170 deaths in 2024 [1]. Thyroid cancer ranks ninth in global cancer incidence [2]. Thyroid cancer exhibits a distinct gender predilection, with females accounting for approximately 75% of all cases [2, 3]. Additionally, the disease can occur across a wide age spectrum, though the median age at diagnosis is the early 50s. Notably, thyroid cancer is the most prevalent malignancy among adolescents and young adults aged 16–33 years [4]. Approximately 84% of thyroid cancer cases are papillary thyroid cancer (PTC), with an estimated 5-year survival of 98.5% [5]. However, aggressive variants of PTC and advanced TNM stage can still threaten the patients' quality of life. Thirty percent of thyroid cancers will not carry any of the known mutations involved in thyroid cancer initiation and progression *(RAS*, *RET/PTC*, or *BRAF (V600E)* mutations) [6]. Thus, it is important to identify novel molecular pathways associated with the progression and prognosis of thyroid cancer.

Recently, long noncoding RNAs (lncRNAs) have been found to have a key role in cancer development and progression including thyroid cancer. This has led investigators to focus on lncRNAs as diagnostic and prognostic thyroid cancer biomarkers. For example, Gugnoni et al*.* found that Linc00941 was upregulated in PTC, and its expression level was correlated with aggressive features in PTC patients [7]. Furthermore, recent work by Guo et al. revealed that lncRNA-MIAT was overexpressed in PTC tissues and impacted cellular proliferation, migration and invasion by regulating by sponging miR-150-5p [8]. Our previous lncRNA expression microarray profiling study reported that many lncRNAs were aberrantly expressed in PTC tissues. Among them, a novel long intergenic noncoding RNA 02454 (LINC02454) was markedly overexpressed in PTC tissues. Functional studies in PTC cells demonstrated that LINC02454 impacted cellular apoptosis and proliferation. Moreover, we found that high LINC02454 expression level was associated with advanced clinical stage and poor disease-free survival in patients with PTC [9]. Furthermore, Cao et al. demonstrated that upregulation of LINC02454 cis-regulated HMGA2 expression by facilitating CREB1 phosphorylation and nuclear translocation, and HMGA2 promoted LINC02454 expression by binding its promoter, thereby accelerating thyroid carcinoma colony formation, migration, invasion, and EMT [10]. Current research on the functional implications of LINC02454 in malignancies other than PTC is limited. Chen et al. have shown that LINC02454, transcriptionally regulated by EGR1, interacts with the CCT complex to modulate TERT expression, maintaining telomere homeostasis and promoting oncogenesis in head and neck squamous cell carcinoma [11]. In addition, LINC02454 was identified as a crucial regulator of laryngeal squamous cell carcinoma progression, promoting cell proliferation, migration, and invasion. Notably, LINC02454 protected laryngeal squamous cell carcinoma cells from copper-induced mitochondrial damage, suggesting its potential as a therapeutic target [12]. However, the possible mechanism of LINC02454 in PTC remains unknown.

Weighted correlation network analysis (WGCNA) can be used for finding clusters (modules) of highly correlated genes, for summarizing such clusters using the module eigengene or an intramodular hub gene (genes with high connectivity in the module), for relating modules to one another and to external sample traits (using eigengene network methodology), and for calculating module membership measures. Correlation networks facilitate network-based gene screening methods that can be used to identify candidate biomarkers or therapeutic targets. These methods have been successfully applied in various biological contexts, e.g. cancer, mouse genetics, yeast genetics, and analysis of brain imaging data. Thus, in the present study, we sought to further explore how the key LINC02454-related pathways and key genes are involved in thyroid cancer oncogenesis by weighted gene coexpression network analysis (WGCNA).

## Material and methods

### Data source and preprocessing

Thyroid cancer RNA sequence expression data together with clinical feature data were obtained from The Cancer Genome Atlas database (TCGA, https://portal.gdc.cancer.gov/). The raw sequencing data were normalized using the voom function of the limma package in R. GSE150899 was collected from GEO (https://www.ncbi.nlm.nih.gov/geo/) to validate the differential expression of five key genes.

### WGCNA network construction and module identification

The “WGCNA” R package was used to explore thyroid cancer-related modules with the dataset obtained from TCGA through the following steps: (i) The median absolute deviation (MAD) in the expression level of each gene was calculated, top 5000 genes with highest MAD were selected for further analysis. (ii) The hclust function of R was used to perform cluster analysis on these samples. (iii) Soft-thresholding was performed for network topology construction, and the appropriate power value was used to construct the network. (iv) Hierarchical clustering and the dynamic tree cutting algorithm were used to detect gene coexpression modules, which were given different colors for visualization. (v) Module-trait associations were examined using the Pearson correlation between the module eigengene and the clinical traits.

### Functional and pathway enrichment analysis

The clusterProfiler R package was utilized to perform Gene Ontology (GO) and Kyoto Encyclopedia of Genes and Genomes (KEGG) pathway enrichment analysis of candidate genes obtained from WGCNA. Pathways with *q* value < 0.2 and *p* value < 0.05 were defined as significantly enriched.

### Co-expressed genes network construction and key gene identification

The coexpressed genes pairs in modules were filter with threshold of 0.1 according to weight. The coexpressed network was constructed and visualized by Cytoscape software (version 3.6.1). Hub genes were identified by MCC topological analysis methods using the Cytoscape plugin cytoHubba. The key genes were defined as the intersection among hub genes identified by MCC in whole coexpression network, LINC02454 coexpressed genes in network and genes in top10 significantly enriched KEGG pathways.

### Validation of key genes in the GEPIA database and GEO dataset

The online database Gene Expression Profiling Interactive Analysis (GEPIA, http://gepia.cancer-pku.cn/) was used to compare expression levels of key genes between thyroid cancer samples and normal samples based on TCGA normal and GTEx data. ANOVA was used to analyze the differentially expressed genes, which were those with |log2FC| values > 1 and q values < 0.01. GEPIA was also applied to explore the prognostic value of key genes though disease-free survival (DFS) curves using the Kaplan‒Meier method with a 50% (median) cutoff and were compared by the log rank test. *P* values < 0.05 were considered statistically significant. The expression level of key genes was also checked in GEO dataset GSE150899.

All statistical analyses in this article were performed using R (4.1.10) and relevant R packages. *P* < 0.05 was considered significant. All asterisks in the text indicate significance, with the corresponding relationships being *p* < 0.0001****, *p* < 0.001***, *p* < 0.01**, *p* < 0.05 *, and *p* > 0.05 ns (not significant).

## Results

### Data collection

**Fig. 1:**
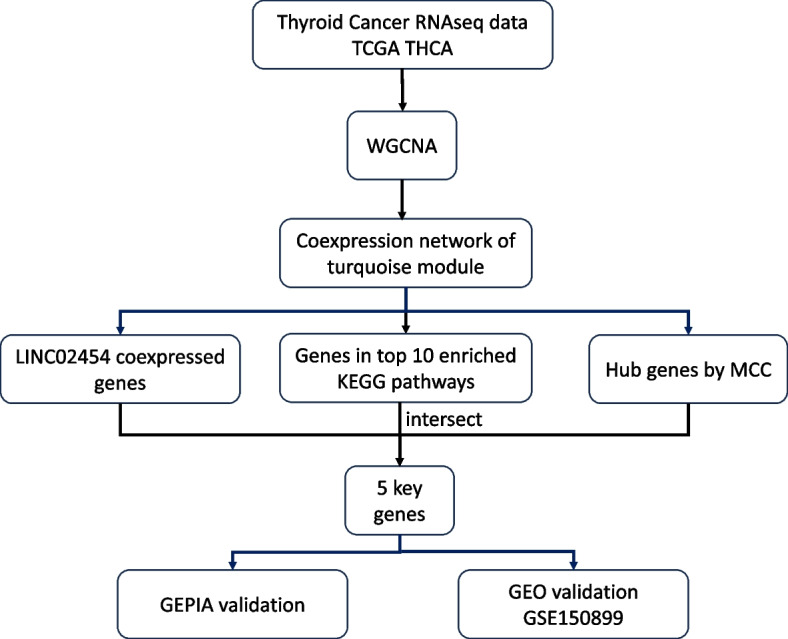
Flow chart of this study design

The strategy of bioinformatics analysis is performed as shown in Fig. 1. A total of 502 thyroid cancer samples were obtained from the TCGA database. Clinical information on thyroid cancer samples, such as sex, race, age, clinical stage and patient survival, was also complied. The boxplot displayed the normalized expression profile of 502 thyroid cancer samples (Supplementary Fig. 1). GEO dataset GSE150899 was utilized to validate the expression of 5 key genes.

### WGCNA network construction

**Fig. 2:**
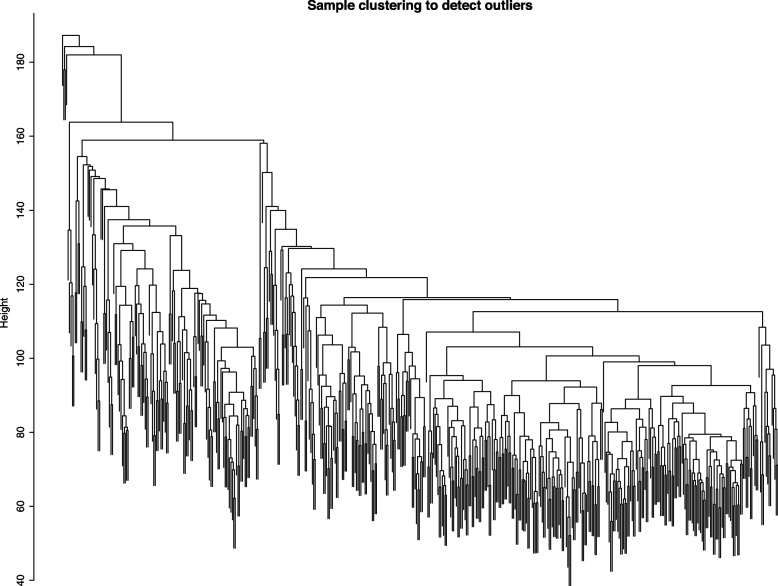
Sample clustering to detect outliers. All the samples were in the clusters, all samples have passed the cuts

**Fig. 3:**
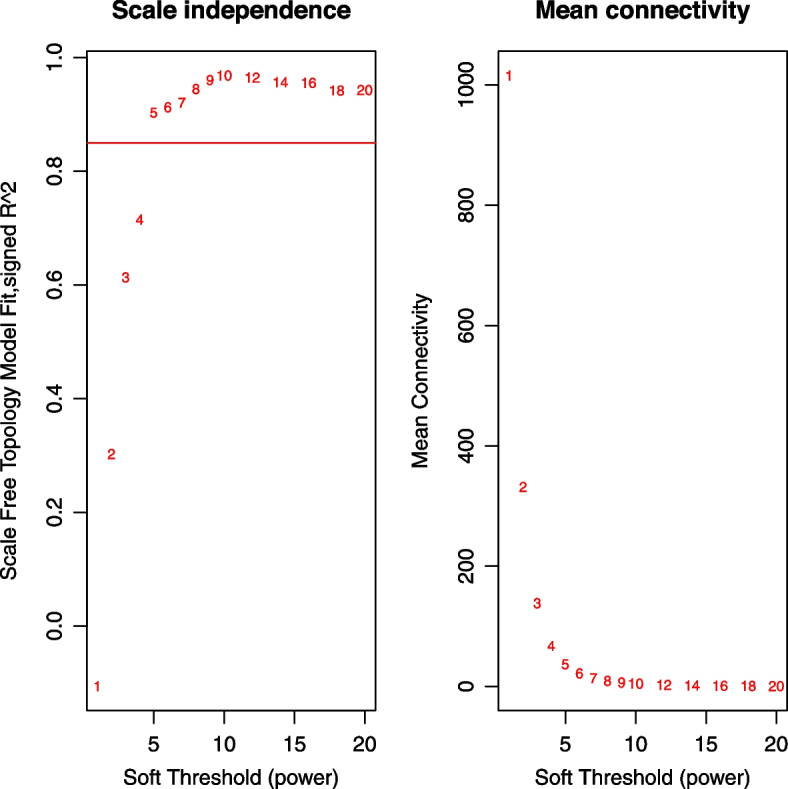
Analysis of network topology for various soft-threshold powers. The left panel shows the function of soft-threshold power on the scale-free topology fit index; the right panel displays the function of soft-threshold power on the mean connectivity

**Fig. 4:**
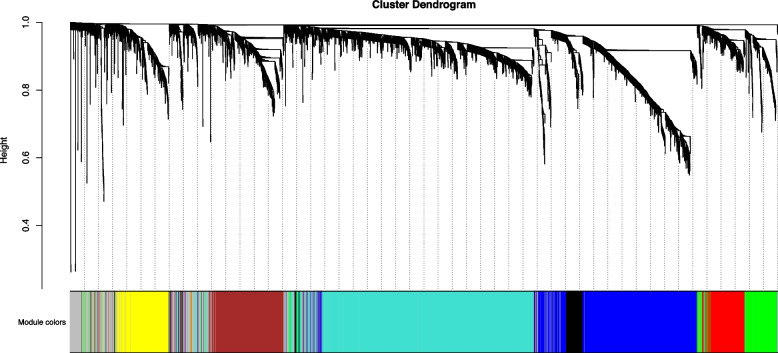
Clustering dendrograms and color display of the co-expression network modules constructed by dissimilarity based on topological overlap. The dynamic tree cut yielded eight modules. Module colors are shown correspondingly

The top 5000 genes with highest MAD were used to conduct WGCNA. To ensure that the identified coexpression module were reliable, hierarchical cluster analysis was performed to remove potential outliers. As shown in Fig. 2, there was no abnormal sample. The scale free topology index achieved 0.85 when soft threshold power β was set to 5, and there was a relatively high average connectivity (Fig. 3). Thus, the constructed network conformed to the power-law distribution and was close to the real biological network. Finally, gene dendrograms were obtained using average linkage hierarchical clustering. The dynamic tree cut yielded eight modules with corresponding colors (Fig. 4). The gene number per module and corresponding colors are shown in Table 1.

**Table 1:** The number of genes in the eight modules

Module colors	Frequency
Turquoise	1619
Blue	1040
Grey	612
Brown	532
Yellow	365
Green	324
Red	316
Black	192

### Key module identification

**Fig. 5:**
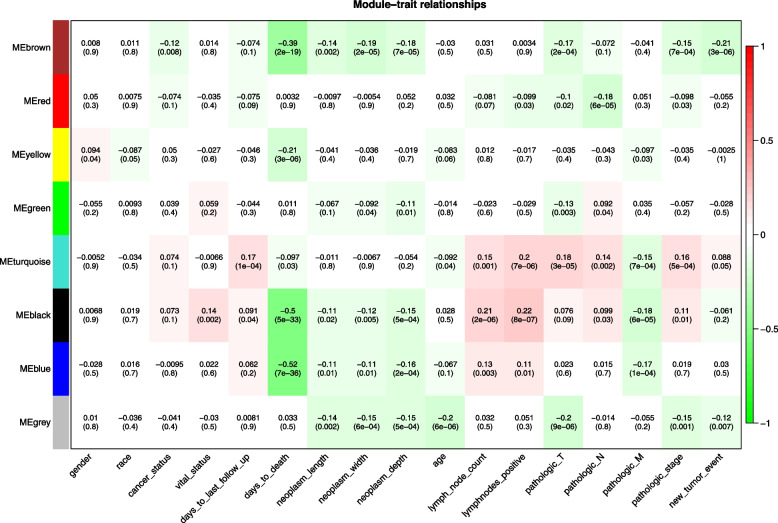
Heatmap of module-trait relationships displaying correlations between module eigengenes and clinical characteristics. Numbers in the table correspond to the correlation coefficient and the p-value in bracket. The degree of correlation is illustrated with the color legend

To search for the most clinically relevant module, the relationships between the module eigengene and the clinical characteristics were depicted in a heat map (Fig. 5). It has been shown that the turquoise module was significantly correlated with the TMN stage, pathologic stage, lymph node count and positive lymph nodes of thyroid cancer. Table 1 showed that the turquoise module consisted of most genes (1619). LINC02454 was right in the turquoise (Supplementary Table 1). Therefore, the turquoise module was selected for further functional enrichment analysis.

### Key module functional enrichment analysis

**Fig. 6:**
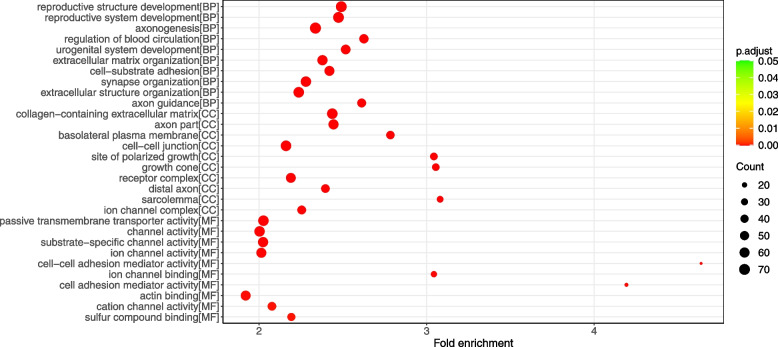
Gene Ontology enrichment of gene clusters involved in the turquoise module regarding biological process, cellular component, and molecular function. The colored dots represent term enrichment: green indicates low significance, red indicates high significance. The size of the dots represents the number of genes in each Gene Ontology term

**Fig. 7:**
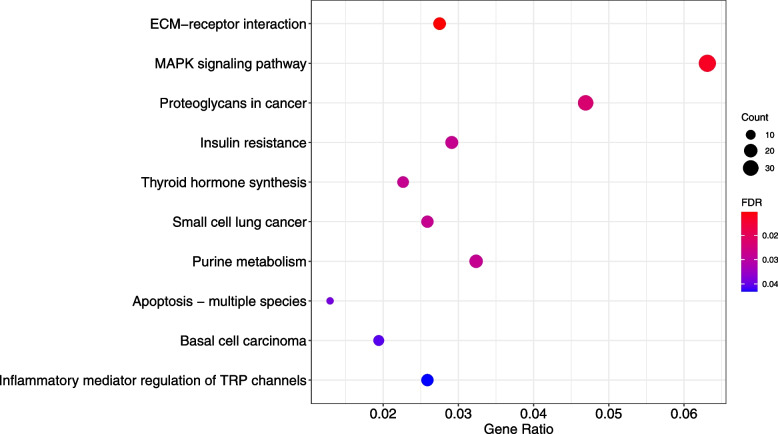
Kyoto Encyclopedia of Genes and Genomes pathways enrichment analysis of gene clusters involved in the turquoise module. The colored dots represent term enrichment: blue indicates low significance, red indicates high significance. The size of the dots represents the number of genes in each Kyoto Encyclopedia of Genes and Genomes pathway

A total of 1619 genes in the turquoise module were subjected to functional enrichment analyses. The top ten terms were selected for visualization. GO analysis indicated that the genes in the turquoise module were mainly enriched in reproductive structure development under biological processes, collagen − containing extracellular matrix under cellular component, and passive transmembrane transporter activity under molecular function (Fig. 6). The results of KEGG pathway analysis showed that the turquoise module regulated pathways included ECM − receptor interaction, MAPK signaling pathway, and proteoglycans in cancer (Fig. 7). Therefore, turquoise module may be associated with thyroid tumorigenesis.

### Key gene identification

**Fig. 8:**
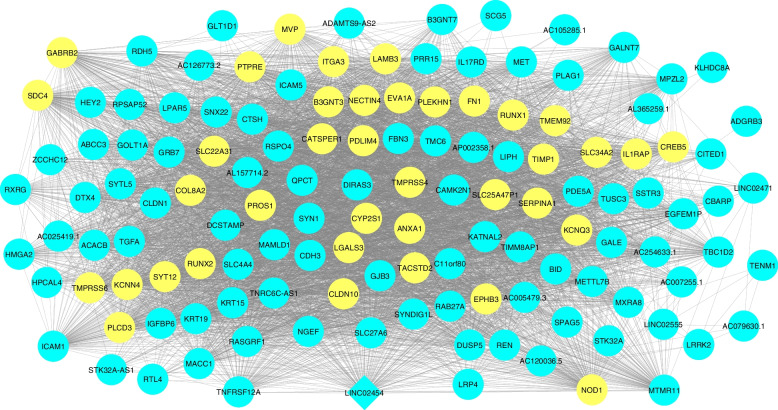
Co-expression network of 131 LINC02454-coexpressed gene. Intersection of top 40 hub genes and genes in top 10 enriched KEGG pathways are highlighted in yellow. LINC02454 is shaped in diamond

To investigate the role of LINC02454 in thyroid cancer tumorigenesis, we constructed coexpression gene network for turquoise module. The network consisted of 1460 nodes and 61,672 edges (Supplementary Fig. 2), which included 131 LINC02454-coexpressed genes (Supplementary Table 2, Fig. 8). 5 key genes (*FN1, LAMB3, ITGA3, SDC4,* and *IL1RAP*) were identified by intersecting these 131 genes with the top 40 hub genes from the network (Supplementary Table 3) identified by MCC method and genes in top 10 enriched KEGG pathways (Supplementary Table 4). Thus, these 5 key genes could be associated with the pathology of PTC and were selected for further analysis.

### Key genes expression level and survival analysis

**Fig. 9:**
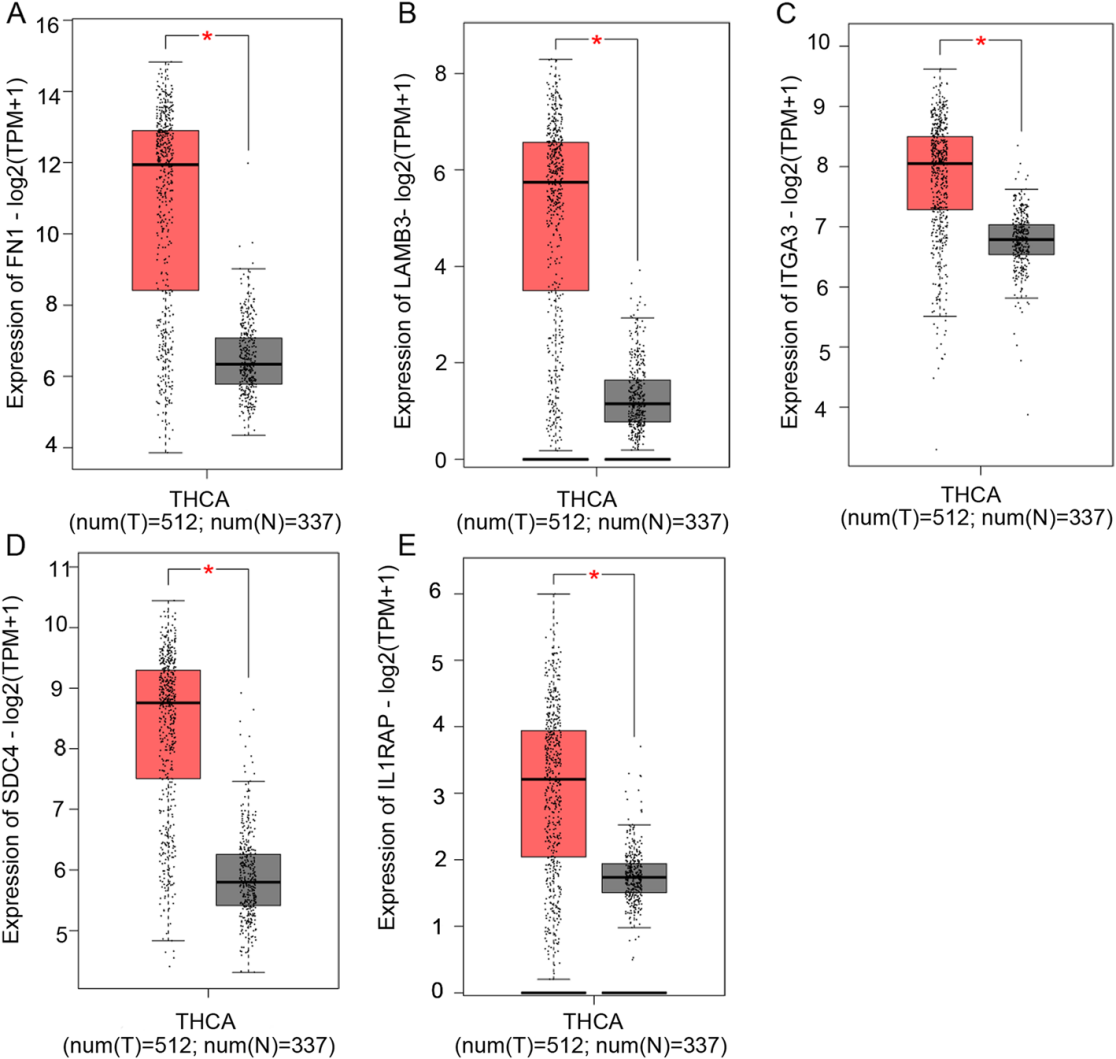
The relative expression of 5 hub genes in thyroid cancer by GEPIA. **A** *FN1*. **B** *LAMB3*. **C** *ITGA3*. **D** *SDC4*. **E** *IL1RAP*. All 5 key genes were significantly upregulated in 512 PTC samples compared with 337normal thyroid samples. Red plots indicate tumor tissue and gray plots indicate normal tissue. *p < 0.01

**Fig. 10:**
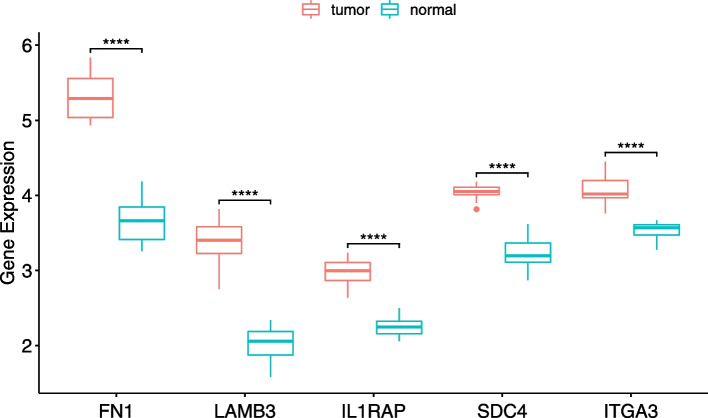
The relative expression of 5 hub genes in thyroid cancer in GSE150899. All 5 key genes were significantly upregulated in PTC samples compared with normal thyroid samples. Red indicates tumor tissue and blue indicates normal tissue. ****p < 0.0001

**Fig. 11:**
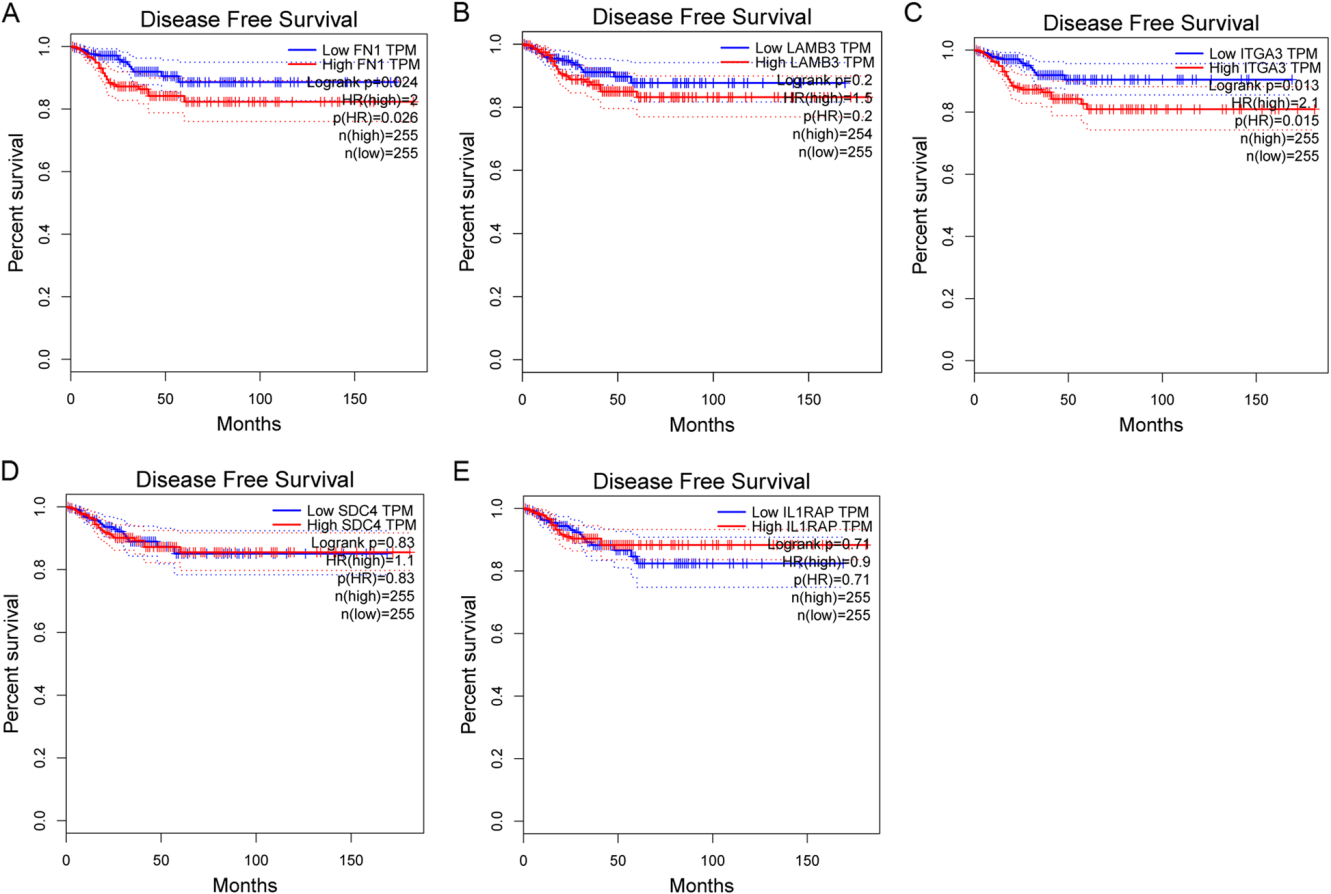
Prognostic value of 5 hub gene expression levels using GEPIA in thyroid cancer. **A** *FN1*. **B** *LAMB3*. **C** *ITGA3*. **D** *SDC4*. **E** *IL1RAP*. Patients with high expression of *FN1* demonstrated significantly shorter DFS than those with low level of *FN1* expression (*P* = 0.024). Patients with high expression of *ITGA3* demonstrated significantly shorter DFS than those with low level of *ITGA3* expression (*P* = 0.013). No significant association was observed between the other three hub genes and DFS. Survival curves were compared using log-rank test. DFS, disease-free survival

Based on the data from GEPIA and TCGA, we found that fibronectin 1 (*FN1*), laminin subunit beta 3 (*LAMB3*), integrin subunit alpha 3 (*ITGA3*), syndecan-4 (*SDC4*), and IL1 receptor-associated protein (*IL1RAP*) were significantly overexpressed in thyroid cancer tissues compared with normal thyroid tissues (*P* < 0.01) (Fig. 9). The aberrant expression of these five key genes were also seen in GEO dataset GSE150899 (Fig. 10). The prognostic value of the 5 key genes were further investigated. As shown in Fig. 11A, PTC patients with high *FN1* expression had significantly shorter DFS than those with low *FN1* expression (*P* < 0.05). Likewise, high ITGA3 expression in PTC patients was associated with poor DFS (*P* < 0.05) (Fig. 9C). No significant association was observed between the other three key genes and disease-free survival (*P* > 0.05) (Fig. 11B, D and E).

## Discussion

In the present study, we discovered the potential pathways and LINC02454-coexpressed genes via WGCNA, giving some clues about the underlying mechanisms of LINC02454 in PTC. The ECM − receptor interaction and MAPK signaling pathway were considered the most significant potential key pathways. We found 131 LINC02454-coexpressed genes, of which FN1, LAMB3, ITGA3, SDC4, and IL1RAP were identified as key genes. Using the online GEPIA database, 5 key genes showed significantly higher expression levels in thyroid cancer tissues than in normal thyroid tissues, which was also confirmed with GEO dataset. More importantly, of these 5 genes, high FN1 and ITGA3 expression was negatively associated with DFS, indicating that FN1 and ITGA3 are considered poor prognostic indicators for PTC. Together, these findings indicate that 5 key genes could affect the ECM-receptor interaction and MAPK signaling pathway to promote PTC development.

The extracellular matrix (ECM) is a complex noncellular unit that is composed of different extracellular glycoproteins. Matrix components can directly interact with cellular receptors to regulate the proliferation, adhesion, migration, differentiation and metastasis of cells. Recent studies have shown that ECM-receptor interaction pathways play a crucial role in promoting tumorigenesis and metastasis in thyroid cancer and other human cancers [13–16]. The genes involved in ECM–receptor interaction pathways are frequently deregulated in cancer patients. Through GO analysis, we found that the differentially expressed genes were mainly enriched in the biological processes of extracellular structure organization, extracellular matrix organization and cell − substrate adhesion, indicating ECM-receptor interaction as a key pathway in thyroid carcinogenesis. *FN1*, a member of the glycoprotein family in the extracellular matrix, has been reported to regulate a variety of biological processes, including cell adhesion, migration and cell movement, in various malignant tumors. For example, a resent study reported that silencing FN1 inhibits YAP1/Hippo pathway activation by enhancing YAP1 phosphorylation, reduces aspartate uptake and utilization via SLC1A3, and suppresses breast cancer cell proliferation, invasion, migration and promotes apoptosis [17]. Another report indicated that the high expression value of *FN1* was associated with significantly poor survival in pancreatic cancer [18]. Moreover, Jiang et al. reported that FN1 was upregulated in papillary PTC and regulated thyroid cancer cell migration, invasion and EMT. Mechanistically, depletion of FN1 rescued the effects of miR-142-3p inhibitor on cell proliferation, invasion, apoptosis and EMT by inactivating the FAK/ERK/PI3K signaling pathway [19]. Additionally, according to Nieto et al*.*’s research, *FN1* was significantly upregulated in recurrent thyroid cancer and impacted cellular migration, highlighting it as a potential prognostic biomarker [20]. In agreement with the above, we found that *FN1* was significantly upregulated in PTC. *FN1* was the LINC02454-coexpressed gene that is involved in the ECM − receptor interaction pathway. More importantly, *FN1* was also a significant predictor of DFS. Together, these findings indicate that *FN1* may act as a new candidate prognostic biomarker for PTC patients.

*ITGA3*, belonging to the integrin family, has been reported to interact with extracellular matrix proteins and promote tumor cell proliferation, migration and survival [21]. Huang et al. noted that ITGA3, a critical integrin subunit, was upregulated in pancreatic cancer. High ITGA3 correlated with increased PD-L1, reduced CD8 + T cells, and unfavorable outcomes in patients receiving chemotherapy or immunotherapy [22]. In thyroid cancer, it has been reported that *ITGA3* can govern PTC cell proliferation, invasion and migration and has been associated with recurrence and short survival [23, 24]. Although some functions of *ITGA3* in PTC have been reported, the potential mechanisms of *ITGA3* in PTC are still unknown. Consistent with the findings in those cancer types, we found that *ITGA3* was highly expressed in PTC tissue, and patients with high *ITGA3* expression showed a significantly poorer DFS. Through WGCNA and KEGG pathway analysis, we showed that *ITGA3* was involved in the ECM–receptor interaction pathway associated with the poor prognosis of PTC patients.

Laminins are large extracellular glycoproteins comprised of three covalently linked chains (α, β, and γ) and are involved in several important biological processes of cellular differentiation, migration, adhesion, proliferation, and survival. *LAMB3* encodes one of the three subunits of LM-332, an extracellular matrix protein secreted by cultured human keratinocytes. A number of studies have revealed that *LAMB3* is involved in cellular invasion and metastasis processes in several tumor types. For example, recent research by Zhang et al. demonstrated that LAMB3 was overexpressed in cervical cancer and promoted cancer cell migration, invasion and survival via the PI3K-AKT pathway [25]. Other researchers found that a combined signature of high LAMA3, LAMB3, and LAMC2 expression was a stronger predictor of poor prognosis in pancreatic adenocarcinoma than individual genes [26]. In thyroid cancer, Jung et al*.* found that *LAMB3* was upregulated in PTC tissues and that loss of *LAMB3* in PTC cells reduced cell migration and invasion via downregulation of epithelial‒mesenchymal transition-associated proteins and inhibition of matrix metalloproteinase 9 [27]. However, the detailed functions of *LAMB3* in PTC are still unclear. In our study, mechanistic exploration revealed that *LAMB3* was a LINC02454-coexpressed gene related to the ECM-receptor interaction pathway in thyroid carcinogenesis.

SDC4 is an important member of the family of transmembrane heparan sulfate proteoglycans, and plays a major role in the interactions between the extracellular matrix and the cell surface [28]. Studies have indicated that the function of *SDC4* is highly associated with human tumorigenesis and development such as hepatocellular cancer, colorectal cancer, and ovarian cancer [29–31]. To our knowledge, there are few reports about the function of *SDC4* in the PTC. A study showed that *SDC4* gene silencing in PTC cells could suppress cell migration and invasion and promote cell apoptosis by inhibiting the activation of the Wnt/β -catenin signaling pathway [32]. These studies demonstrated that *SDC4* could be considered as a tumor promoter in human cancers. However, studies on the precise molecular mechanisms study of *SDC4* in PTC are still limited. In our study, *SDC4* expression was significantly increased in PTC tissues. Additionally, *SDC4* might be coexpressed with LINC02454 and correlated with the ECM-receptor interaction pathway in PTC.

The MAPK signaling pathway plays a vital role in several processes of tumorigenesis such as tumor cell proliferation, migration, and invasion in various cancers including thyroid cancer. IL1RAP plays a crucial role in inflammation through the IL-1, IL-33, and IL-36 signaling pathways. Importantly, IL1RAP is overexpressed on tumor cells across various cancers, supporting its potential involvement in carcinogenesis. Structurally, IL1RAP consists of an extracellular cytokine-binding domain and an intracellular signaling domain capable of activating inflammatory pathways like MAPK and NF-κB pathways [33]. Zhang et al*.* noted that high *IL1RAP* expression in Ewing sarcoma may drive Ewing sarcoma progression by mediating local invasion and metastatic capacity [34]. Another recent study showed that *IL1RAP* overexpression was associated with worse overall survival in pancreatic cancer patients, which was attributed to its facilitation of tumor cell viability, invasiveness, and clonogenic growth [35]. To date, there are few data about its role in thyroid cancer. Smallridge et al*.* found that *IL1RAP* was upregulated in *BRAF V600E* PTC compared with *BRAF* wild-type patients via RNA sequencing analysis [36]. However, there is currently no clear evidence of the detailed functions of *IL1RAP* in PTC. Our study showed that *IL1RAP* was highly expressed in thyroid cancer tissues compared with normal tissues. Moreover, *IL1RAP* seemed to be coexpressed with LINC02454 and activated the MAPK pathway in thyroid tumorigenesis.

In this work, we proposed a novel methodology to identify thyroid cancer related key genes, by integrating WGCNA, KEGG enrichment analysis and network analysis. The identified key genes were significantly differentially expressed between tumor and normal samples, and they have been proved to be related to prognosis of thyroid cancer. This method can also be utilized to study other cancers. In previous articles, the analysis usually starts with differential expression analysis, and subsequent analysis is based on genes that show differential expression [37, 38]. However, our article starts with the identification module and then searches for modules closely related to clinical characteristics of thyroid cancer. Furthermore, we integrated three different bioinformatics methods to screen out five key genes. Although these results indicate the underlying mechanism of action of LINC02454 in PTC, there are several limitations to our study. First, there are few functional studies of these candidate genes in PTC cells. Second, our study conducted the LINC02454 centered coexpression network analysis, but whether these candidate genes could be regarded as potential diagnostic and prognostic biomarkers may need further investigation. Third, the precise mechanism by which LINC02454 affects the ECM-receptor interaction and MAPK signaling remains poorly explored.

## Conclusions

We conducted comprehensive bioinformatics analyses to investigate the LINC02454-related key pathways and coexpressed genes in PTC. The expression levels of 5 key genes *(FN1, LAMB3, ITGA3, SDC4,* and *IL1RAP*) were significantly upregulated in PTC tissues. In addition, *FN1* and *ITGA3* were correlated with poor prognosis of PTC. Mechanistic exploration revealed that key genes could affect the ECM-receptor interaction and MAPK signaling pathway in PTC pathogenesis.

### Supplementary Information

Supplementary Material 1: Supplementary Fig. 1. Boxplot to display the expression of normalized data from 502 thyroid cancer samples.

Supplementary Material 2: Supplementary Fig. 2. Co-expression network of all genes in turquoise module.

Supplementary Material 3.

Supplementary Material 4.

Supplementary Material 5.

Supplementary Material 6.

## Data Availability

The datasets used and/or analysed during the current study are available from the corresponding author on reasonable request.

## Abbreviations

LINC02454: Long intergenic noncoding RNA 02454
PTC: Papillary thyroid cancer
WGCNA: Weighted gene coexpression network analysis
KEGG: Kyoto Encyclopedia of Genes and Genomes
GEPIA: The Gene Expression Profiling Interactive Analysis
