# Supplementary figures and images for: Identification of LINC02454-related key pathways and genes in papillary thyroid cancer by weighted gene coexpression network analysis (WGCNA)
Source: Thyroid Res. 2024 Sep 2;17:17. doi: 10.1186/s13044-024-00205-8 (PMC11367880; doi:10.1186/s13044-024-00205-8)

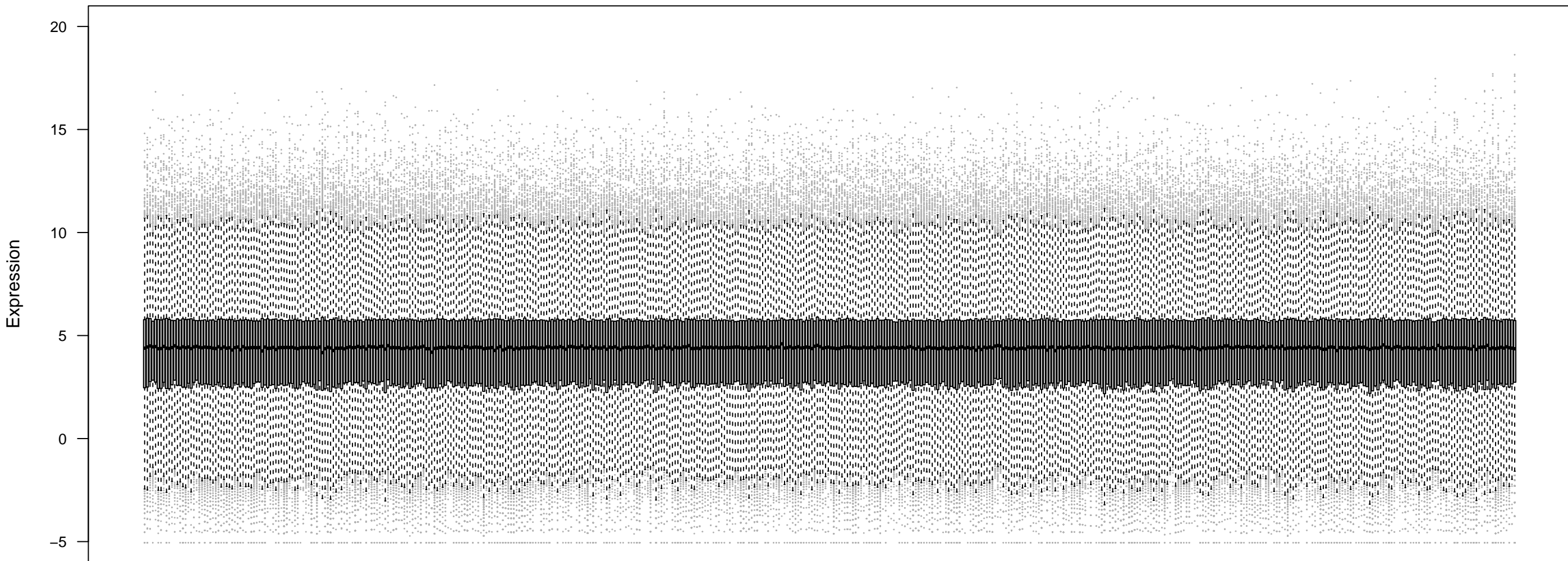

Supplement: Supplementary file 1 — Supplementary Material 1: Supplementary Fig. 1. Boxplot to display the expression of normalized data from 502 thyroid cancer samples. [file 13044_2024_205_MOESM1_ESM.pdf]

LINC02245

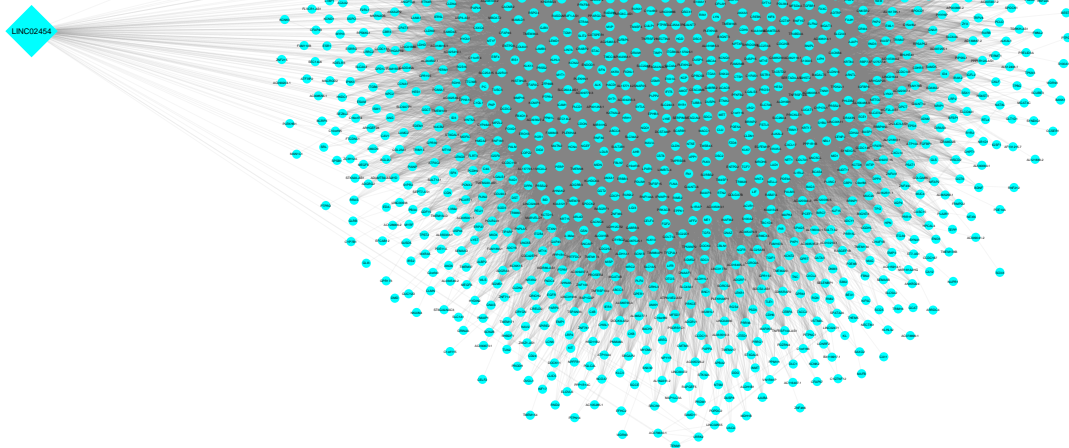

Supplement: Supplementary file 2 — Supplementary Material 2: Supplementary Fig. 2. Co-expression network of all genes in turquoise module. [file 13044_2024_205_MOESM2_ESM.pdf]
