# Supplementary material for: Identification of LINC02454-related key pathways and genes in papillary thyroid cancer by weighted gene coexpression network analysis (WGCNA)
Source: Thyroid Res. 2024 Sep 2;17:17. doi: 10.1186/s13044-024-00205-8 (PMC11367880; doi:10.1186/s13044-024-00205-8)
Supplement: Supplementary file 3 — Supplementary Material 3. [file 13044_2024_205_MOESM3_ESM.docx]

Supplementary Table 1 The name of all genes in the turquoise module

| Ensembl ID | gene symbol | type |
| --- | --- | --- |
| ENSG00000167757 | KLK11 | protein_coding |
| ENSG00000147256 | ARHGAP36 | protein_coding |
| ENSG00000163395 | IGFN1 | protein_coding |
| ENSG00000211452 | DIO1 | protein_coding |
| ENSG00000224568 | LINC01886 | long_non_coding |
| ENSG00000075035 | WSCD2 | protein_coding |
| ENSG00000205038 | PKHD1L1 | protein_coding |
| ENSG00000155897 | ADCY8 | protein_coding |
| ENSG00000086548 | CEACAM6 | protein_coding |
| ENSG00000124205 | EDN3 | protein_coding |
| ENSG00000115602 | IL1RL1 | protein_coding |
| ENSG00000163817 | SLC6A20 | protein_coding |
| ENSG00000125144 | MT1G | protein_coding |
| ENSG00000198535 | C2CD4A | protein_coding |
| ENSG00000166897 | ELFN2 | protein_coding |
| ENSG00000115705 | TPO | protein_coding |
| ENSG00000130226 | DPP6 | protein_coding |
| ENSG00000134762 | DSC3 | protein_coding |
| ENSG00000256870 | SLC5A8 | protein_coding |
| ENSG00000117069 | ST6GALNAC5 | protein_coding |
| ENSG00000275896 | PRSS2 | protein_coding |
| ENSG00000165966 | PDZRN4 | protein_coding |
| ENSG00000145824 | CXCL14 | protein_coding |
| ENSG00000204544 | MUC21 | protein_coding |
| ENSG00000041353 | RAB27B | protein_coding |
| ENSG00000153993 | SEMA3D | protein_coding |
| ENSG00000205358 | MT1H | protein_coding |
| ENSG00000067715 | SYT1 | protein_coding |
| ENSG00000109255 | NMU | protein_coding |
| ENSG00000172020 | GAP43 | protein_coding |
| ENSG00000233705 | SLC26A4-AS1 | long_non_coding |
| ENSG00000165495 | PKNOX2 | protein_coding |
| ENSG00000160180 | TFF3 | protein_coding |
| ENSG00000128422 | KRT17 | protein_coding |
| ENSG00000251002 | AC244502.1 | long_non_coding |
| ENSG00000006016 | CRLF1 | protein_coding |
| ENSG00000169035 | KLK7 | protein_coding |
| ENSG00000075461 | CACNG4 | protein_coding |
| ENSG00000145087 | STXBP5L | protein_coding |
| ENSG00000134873 | CLDN10 | protein_coding |
| ENSG00000204866 | IGFL2 | protein_coding |
| ENSG00000006071 | ABCC8 | protein_coding |
| ENSG00000133048 | CHI3L1 | protein_coding |
| ENSG00000148346 | LCN2 | protein_coding |
| ENSG00000187045 | TMPRSS6 | protein_coding |
| ENSG00000137440 | FGFBP1 | protein_coding |
| ENSG00000072315 | TRPC5 | protein_coding |
| ENSG00000196353 | CPNE4 | protein_coding |
| ENSG00000167105 | TMEM92 | protein_coding |
| ENSG00000100341 | PNPLA5 | protein_coding |
| ENSG00000137648 | TMPRSS4 | protein_coding |
| ENSG00000259459 | AC087525.1 | long_non_coding |
| ENSG00000124493 | GRM4 | protein_coding |
| ENSG00000102924 | CBLN1 | protein_coding |
| ENSG00000254799 | SLC25A47P1 | pseudogene |
| ENSG00000161509 | GRIN2C | protein_coding |
| ENSG00000186417 | GLDN | protein_coding |
| ENSG00000166426 | CRABP1 | protein_coding |
| ENSG00000214797 | AP002358.1 | long_non_coding |
| ENSG00000171877 | FRMD5 | protein_coding |
| ENSG00000166589 | CDH16 | protein_coding |
| ENSG00000237463 | AL157714.2 | long_non_coding |
| ENSG00000124107 | SLPI | protein_coding |
| ENSG00000101197 | BIRC7 | protein_coding |
| ENSG00000189377 | CXCL17 | protein_coding |
| ENSG00000115194 | SLC30A3 | protein_coding |
| ENSG00000164935 | DCSTAMP | protein_coding |
| ENSG00000102109 | PCSK1N | protein_coding |
| ENSG00000125798 | FOXA2 | protein_coding |
| ENSG00000165188 | RNF183 | protein_coding |
| ENSG00000184905 | TCEAL2 | protein_coding |
| ENSG00000179913 | B3GNT3 | protein_coding |
| ENSG00000109182 | CWH43 | protein_coding |
| ENSG00000157765 | SLC34A2 | protein_coding |
| ENSG00000156414 | TDRD9 | protein_coding |
| ENSG00000074410 | CA12 | protein_coding |
| ENSG00000130720 | FIBCD1 | protein_coding |
| ENSG00000203585 | LINC02408 | long_non_coding |
| ENSG00000147689 | FAM83A | protein_coding |
| ENSG00000163362 | INAVA | protein_coding |
| ENSG00000138316 | ADAMTS14 | protein_coding |
| ENSG00000105696 | TMEM59L | protein_coding |
| ENSG00000080493 | SLC4A4 | protein_coding |
| ENSG00000169903 | TM4SF4 | protein_coding |
| ENSG00000166292 | TMEM100 | protein_coding |
| ENSG00000115414 | FN1 | protein_coding |
| ENSG00000152128 | TMEM163 | protein_coding |
| ENSG00000204421 | LY6G6C | protein_coding |
| ENSG00000169282 | KCNAB1 | protein_coding |
| ENSG00000166448 | TMEM130 | protein_coding |
| ENSG00000267206 | LCN6 | protein_coding |
| ENSG00000236699 | ARHGEF38 | protein_coding |
| ENSG00000187017 | ESPN | protein_coding |
| ENSG00000136542 | GALNT5 | protein_coding |
| ENSG00000169594 | BNC1 | protein_coding |
| ENSG00000138308 | PLA2G12B | protein_coding |
| ENSG00000223813 | AC007255.1 | long_non_coding |
| ENSG00000185352 | HS6ST3 | protein_coding |
| ENSG00000175793 | SFN | protein_coding |
| ENSG00000198797 | BRINP2 | protein_coding |
| ENSG00000227036 | LINC00511 | long_non_coding |
| ENSG00000228789 | HCG22 | long_non_coding |
| ENSG00000137699 | TRIM29 | protein_coding |
| ENSG00000135925 | WNT10A | protein_coding |
| ENSG00000091137 | SLC26A4 | protein_coding |
| ENSG00000173227 | SYT12 | protein_coding |
| ENSG00000229116 | AL137026.1 | long_non_coding |
| ENSG00000154319 | FAM167A | protein_coding |
| ENSG00000129451 | KLK10 | protein_coding |
| ENSG00000066382 | MPPED2 | protein_coding |
| ENSG00000099812 | MISP | protein_coding |
| ENSG00000196549 | MME | protein_coding |
| ENSG00000116299 | KIAA1324 | protein_coding |
| ENSG00000178602 | OTOS | protein_coding |
| ENSG00000175315 | CST6 | protein_coding |
| ENSG00000113924 | HGD | protein_coding |
| ENSG00000164199 | ADGRV1 | protein_coding |
| ENSG00000165376 | CLDN2 | protein_coding |
| ENSG00000073282 | TP63 | protein_coding |
| ENSG00000131094 | C1QL1 | protein_coding |
| ENSG00000116983 | HPCAL4 | protein_coding |
| ENSG00000132329 | RAMP1 | protein_coding |
| ENSG00000117152 | RGS4 | protein_coding |
| ENSG00000149294 | NCAM1 | protein_coding |
| ENSG00000130203 | APOE | protein_coding |
| ENSG00000185567 | AHNAK2 | protein_coding |
| ENSG00000205502 | C2CD4B | protein_coding |
| ENSG00000104059 | FAM189A1 | protein_coding |
| ENSG00000130054 | FAM155B | protein_coding |
| ENSG00000162552 | WNT4 | protein_coding |
| ENSG00000166206 | GABRB3 | protein_coding |
| ENSG00000149654 | CDH22 | protein_coding |
| ENSG00000134548 | SPX | protein_coding |
| ENSG00000133169 | BEX1 | protein_coding |
| ENSG00000157502 | MUM1L1 | protein_coding |
| ENSG00000179242 | CDH4 | protein_coding |
| ENSG00000128268 | MGAT3 | protein_coding |
| ENSG00000197046 | SIGLEC15 | protein_coding |
| ENSG00000107742 | SPOCK2 | protein_coding |
| ENSG00000111863 | ADTRP | protein_coding |
| ENSG00000174938 | SEZ6L2 | protein_coding |
| ENSG00000168481 | LGI3 | protein_coding |
| ENSG00000113396 | SLC27A6 | protein_coding |
| ENSG00000185499 | MUC1 | protein_coding |
| ENSG00000171885 | AQP4 | protein_coding |
| ENSG00000155980 | KIF5A | protein_coding |
| ENSG00000146555 | SDK1 | protein_coding |
| ENSG00000260943 | LINC02555 | long_non_coding |
| ENSG00000204632 | HLA-G | protein_coding |
| ENSG00000187823 | RTL4 | protein_coding |
| ENSG00000153707 | PTPRD | protein_coding |
| ENSG00000147255 | IGSF1 | protein_coding |
| ENSG00000081041 | CXCL2 | protein_coding |
| ENSG00000064300 | NGFR | protein_coding |
| ENSG00000161798 | AQP5 | protein_coding |
| ENSG00000054803 | CBLN4 | protein_coding |
| ENSG00000104783 | KCNN4 | protein_coding |
| ENSG00000041982 | TNC | protein_coding |
| ENSG00000105695 | MAG | protein_coding |
| ENSG00000135373 | EHF | protein_coding |
| ENSG00000147606 | SLC26A7 | protein_coding |
| ENSG00000241644 | INMT | protein_coding |
| ENSG00000272482 | AC254633.1 | long_non_coding |
| ENSG00000078725 | BRINP1 | protein_coding |
| ENSG00000258947 | TUBB3 | protein_coding |
| ENSG00000113946 | CLDN16 | protein_coding |
| ENSG00000198626 | RYR2 | protein_coding |
| ENSG00000133710 | SPINK5 | protein_coding |
| ENSG00000174460 | ZCCHC12 | protein_coding |
| ENSG00000198417 | MT1F | protein_coding |
| ENSG00000149403 | GRIK4 | protein_coding |
| ENSG00000196368 | NUDT11 | protein_coding |
| ENSG00000184613 | NELL2 | protein_coding |
| ENSG00000133101 | CCNA1 | protein_coding |
| ENSG00000123689 | G0S2 | protein_coding |
| ENSG00000012124 | CD22 | protein_coding |
| ENSG00000183317 | EPHA10 | protein_coding |
| ENSG00000069812 | HES2 | protein_coding |
| ENSG00000050767 | COL23A1 | protein_coding |
| ENSG00000176532 | PRR15 | protein_coding |
| ENSG00000138061 | CYP1B1 | protein_coding |
| ENSG00000141639 | MAPK4 | protein_coding |
| ENSG00000113389 | NPR3 | protein_coding |
| ENSG00000179593 | ALOX15B | protein_coding |
| ENSG00000150051 | MKX | protein_coding |
| ENSG00000130513 | GDF15 | protein_coding |
| ENSG00000259264 | NA | NA |
| ENSG00000134668 | SPOCD1 | protein_coding |
| ENSG00000058335 | RASGRF1 | protein_coding |
| ENSG00000136689 | IL1RN | protein_coding |
| ENSG00000113083 | LOX | protein_coding |
| ENSG00000205364 | MT1M | protein_coding |
| ENSG00000129596 | CDO1 | protein_coding |
| ENSG00000183783 | KCTD8 | protein_coding |
| ENSG00000152463 | OLAH | protein_coding |
| ENSG00000196260 | SFTA2 | protein_coding |
| ENSG00000131771 | PPP1R1B | protein_coding |
| ENSG00000158022 | TRIM63 | protein_coding |
| ENSG00000082482 | KCNK2 | protein_coding |
| ENSG00000109321 | AREG | protein_coding |
| ENSG00000109819 | PPARGC1A | protein_coding |
| ENSG00000267128 | RNF157-AS1 | long_non_coding |
| ENSG00000101098 | RIMS4 | protein_coding |
| ENSG00000167741 | GGT6 | protein_coding |
| ENSG00000270547 | LINC01235 | long_non_coding |
| ENSG00000164099 | PRSS12 | protein_coding |
| ENSG00000137868 | STRA6 | protein_coding |
| ENSG00000174939 | ASPHD1 | protein_coding |
| ENSG00000115112 | TFCP2L1 | protein_coding |
| ENSG00000010282 | HHATL | protein_coding |
| ENSG00000260807 | AC009041.2 | long_non_coding |
| ENSG00000064218 | DMRT3 | protein_coding |
| ENSG00000157404 | KIT | protein_coding |
| ENSG00000184292 | TACSTD2 | protein_coding |
| ENSG00000126752 | SSX1 | protein_coding |
| ENSG00000092758 | COL9A3 | protein_coding |
| ENSG00000144681 | STAC | protein_coding |
| ENSG00000125931 | CITED1 | protein_coding |
| ENSG00000108924 | HLF | protein_coding |
| ENSG00000272384 | AC016405.3 | long_non_coding |
| ENSG00000114270 | COL7A1 | protein_coding |
| ENSG00000156103 | MMP16 | protein_coding |
| ENSG00000165125 | TRPV6 | protein_coding |
| ENSG00000175294 | CATSPER1 | protein_coding |
| ENSG00000150625 | GPM6A | protein_coding |
| ENSG00000138829 | FBN2 | protein_coding |
| ENSG00000196407 | THEM5 | protein_coding |
| ENSG00000145703 | IQGAP2 | protein_coding |
| ENSG00000068976 | PYGM | protein_coding |
| ENSG00000196352 | CD55 | protein_coding |
| ENSG00000181218 | HIST3H2A | protein_coding |
| ENSG00000169908 | TM4SF1 | protein_coding |
| ENSG00000197444 | OGDHL | protein_coding |
| ENSG00000128573 | FOXP2 | protein_coding |
| ENSG00000136928 | GABBR2 | protein_coding |
| ENSG00000168907 | PLA2G4F | protein_coding |
| ENSG00000150722 | PPP1R1C | protein_coding |
| ENSG00000185761 | ADAMTSL5 | protein_coding |
| ENSG00000164171 | ITGA2 | protein_coding |
| ENSG00000225969 | ABHD11-AS1 | pseudogene |
| ENSG00000130164 | LDLR | protein_coding |
| ENSG00000073756 | PTGS2 | protein_coding |
| ENSG00000100253 | MIOX | protein_coding |
| ENSG00000112232 | KHDRBS2 | protein_coding |
| ENSG00000167644 | C19orf33 | protein_coding |
| ENSG00000128655 | PDE11A | protein_coding |
| ENSG00000184156 | KCNQ3 | protein_coding |
| ENSG00000161896 | IP6K3 | protein_coding |
| ENSG00000184226 | PCDH9 | protein_coding |
| ENSG00000173641 | HSPB7 | protein_coding |
| ENSG00000185483 | ROR1 | protein_coding |
| ENSG00000064270 | ATP2C2 | protein_coding |
| ENSG00000196581 | AJAP1 | protein_coding |
| ENSG00000115461 | IGFBP5 | protein_coding |
| ENSG00000123700 | KCNJ2 | protein_coding |
| ENSG00000171346 | KRT15 | protein_coding |
| ENSG00000143839 | REN | protein_coding |
| ENSG00000134020 | PEBP4 | protein_coding |
| ENSG00000158813 | EDA | protein_coding |
| ENSG00000167600 | CYP2S1 | protein_coding |
| ENSG00000156510 | HKDC1 | protein_coding |
| ENSG00000022267 | FHL1 | protein_coding |
| ENSG00000157680 | DGKI | protein_coding |
| ENSG00000009765 | IYD | protein_coding |
| ENSG00000196754 | S100A2 | protein_coding |
| ENSG00000187634 | SAMD11 | protein_coding |
| ENSG00000278195 | SSTR3 | protein_coding |
| ENSG00000114805 | PLCH1 | protein_coding |
| ENSG00000007516 | BAIAP3 | protein_coding |
| ENSG00000118473 | SGIP1 | protein_coding |
| ENSG00000224189 | HAGLR | long_non_coding |
| ENSG00000184254 | ALDH1A3 | protein_coding |
| ENSG00000081479 | LRP2 | protein_coding |
| ENSG00000033122 | LRRC7 | protein_coding |
| ENSG00000182752 | PAPPA | protein_coding |
| ENSG00000103485 | QPRT | protein_coding |
| ENSG00000082175 | PGR | protein_coding |
| ENSG00000136842 | TMOD1 | protein_coding |
| ENSG00000158055 | GRHL3 | protein_coding |
| ENSG00000127472 | PLA2G5 | protein_coding |
| ENSG00000145864 | GABRB2 | protein_coding |
| ENSG00000225342 | AC079630.1 | long_non_coding |
| ENSG00000123901 | GPR83 | protein_coding |
| ENSG00000008056 | SYN1 | protein_coding |
| ENSG00000198947 | DMD | protein_coding |
| ENSG00000166546 | BEAN1 | protein_coding |
| ENSG00000197410 | DCHS2 | protein_coding |
| ENSG00000156049 | GNA14 | protein_coding |
| ENSG00000138166 | DUSP5 | protein_coding |
| ENSG00000174950 | CD164L2 | protein_coding |
| ENSG00000148734 | NPFFR1 | protein_coding |
| ENSG00000185052 | SLC24A3 | protein_coding |
| ENSG00000168754 | FAM178B | protein_coding |
| ENSG00000163435 | ELF3 | protein_coding |
| ENSG00000147041 | SYTL5 | protein_coding |
| ENSG00000144366 | GULP1 | protein_coding |
| ENSG00000270607 | AC009549.1 | long_non_coding |
| ENSG00000132821 | VSTM2L | protein_coding |
| ENSG00000141682 | PMAIP1 | protein_coding |
| ENSG00000156535 | CD109 | protein_coding |
| ENSG00000049323 | LTBP1 | protein_coding |
| ENSG00000146700 | SSC4D | protein_coding |
| ENSG00000101198 | NKAIN4 | protein_coding |
| ENSG00000135069 | PSAT1 | protein_coding |
| ENSG00000265369 | PCAT18 | long_non_coding |
| ENSG00000150995 | ITPR1 | protein_coding |
| ENSG00000155265 | GOLGA7B | protein_coding |
| ENSG00000167779 | IGFBP6 | protein_coding |
| ENSG00000225526 | MKRN2OS | protein_coding |
| ENSG00000197747 | S100A10 | protein_coding |
| ENSG00000050555 | LAMC3 | protein_coding |
| ENSG00000162722 | TRIM58 | protein_coding |
| ENSG00000149970 | CNKSR2 | protein_coding |
| ENSG00000139200 | PIANP | protein_coding |
| ENSG00000261115 | TMEM178B | protein_coding |
| ENSG00000054179 | ENTPD2 | protein_coding |
| ENSG00000003096 | KLHL13 | protein_coding |
| ENSG00000000971 | CFH | protein_coding |
| ENSG00000196878 | LAMB3 | protein_coding |
| ENSG00000081277 | PKP1 | protein_coding |
| ENSG00000138622 | HCN4 | protein_coding |
| ENSG00000155966 | AFF2 | protein_coding |
| ENSG00000171345 | KRT19 | protein_coding |
| ENSG00000042062 | RIPOR3 | protein_coding |
| ENSG00000057294 | PKP2 | protein_coding |
| ENSG00000149948 | HMGA2 | protein_coding |
| ENSG00000176092 | CRYBG2 | protein_coding |
| ENSG00000196639 | HRH1 | protein_coding |
| ENSG00000183091 | NEB | protein_coding |
| ENSG00000169435 | RASSF6 | protein_coding |
| ENSG00000175592 | FOSL1 | protein_coding |
| ENSG00000178222 | RNF212 | protein_coding |
| ENSG00000176046 | NUPR1 | protein_coding |
| ENSG00000275993 | CU639417.2 | protein_coding |
| ENSG00000088992 | TESC | protein_coding |
| ENSG00000135437 | RDH5 | protein_coding |
| ENSG00000113361 | CDH6 | protein_coding |
| ENSG00000104808 | DHDH | protein_coding |
| ENSG00000167767 | KRT80 | protein_coding |
| ENSG00000233680 | HNRNPA1P27 | pseudogene |
| ENSG00000158747 | NBL1 | protein_coding |
| ENSG00000137747 | TMPRSS13 | protein_coding |
| ENSG00000088002 | SULT2B1 | protein_coding |
| ENSG00000101463 | SYNDIG1 | protein_coding |
| ENSG00000124602 | UNC5CL | protein_coding |
| ENSG00000176697 | BDNF | protein_coding |
| ENSG00000261150 | EPPK1 | protein_coding |
| ENSG00000196154 | S100A4 | protein_coding |
| ENSG00000106541 | AGR2 | protein_coding |
| ENSG00000105088 | OLFM2 | protein_coding |
| ENSG00000168772 | CXXC4 | protein_coding |
| ENSG00000146592 | CREB5 | protein_coding |
| ENSG00000101282 | RSPO4 | protein_coding |
| ENSG00000184012 | TMPRSS2 | protein_coding |
| ENSG00000135253 | KCP | protein_coding |
| ENSG00000153446 | C16orf89 | protein_coding |
| ENSG00000152377 | SPOCK1 | protein_coding |
| ENSG00000141052 | MYOCD | protein_coding |
| ENSG00000101311 | FERMT1 | protein_coding |
| ENSG00000235770 | LINC00607 | long_non_coding |
| ENSG00000198805 | PNP | protein_coding |
| ENSG00000109861 | CTSC | protein_coding |
| ENSG00000248323 | LUCAT1 | long_non_coding |
| ENSG00000281162 | AC005035.1 | long_non_coding |
| ENSG00000163638 | ADAMTS9 | protein_coding |
| ENSG00000154102 | C16orf74 | protein_coding |
| ENSG00000185274 | GALNT17 | protein_coding |
| ENSG00000007174 | DNAH9 | protein_coding |
| ENSG00000112541 | PDE10A | protein_coding |
| ENSG00000159648 | TEPP | protein_coding |
| ENSG00000182489 | XKRX | protein_coding |
| ENSG00000279338 | NA | NA |
| ENSG00000225285 | LINC01770 | long_non_coding |
| ENSG00000169083 | AR | protein_coding |
| ENSG00000164761 | TNFRSF11B | protein_coding |
| ENSG00000074706 | IPCEF1 | protein_coding |
| ENSG00000064692 | SNCAIP | protein_coding |
| ENSG00000180801 | ARSJ | protein_coding |
| ENSG00000147234 | FRMPD3 | protein_coding |
| ENSG00000115226 | FNDC4 | protein_coding |
| ENSG00000238271 | IFNWP19 | pseudogene |
| ENSG00000174611 | KY | protein_coding |
| ENSG00000034053 | APBA2 | protein_coding |
| ENSG00000155849 | ELMO1 | protein_coding |
| ENSG00000143320 | CRABP2 | protein_coding |
| ENSG00000152207 | CYSLTR2 | protein_coding |
| ENSG00000092295 | TGM1 | protein_coding |
| ENSG00000187134 | AKR1C1 | protein_coding |
| ENSG00000112559 | MDFI | protein_coding |
| ENSG00000185818 | NAT8L | protein_coding |
| ENSG00000178531 | CTXN1 | protein_coding |
| ENSG00000175920 | DOK7 | protein_coding |
| ENSG00000176406 | RIMS2 | protein_coding |
| ENSG00000261586 | AC068987.4 | long_non_coding |
| ENSG00000136155 | SCEL | protein_coding |
| ENSG00000162490 | DRAXIN | protein_coding |
| ENSG00000268307 | LINC02560 | long_non_coding |
| ENSG00000259803 | SLC22A31 | protein_coding |
| ENSG00000073849 | ST6GAL1 | protein_coding |
| ENSG00000238243 | OR2W3 | protein_coding |
| ENSG00000104892 | KLC3 | protein_coding |
| ENSG00000151136 | BTBD11 | protein_coding |
| ENSG00000171236 | LRG1 | protein_coding |
| ENSG00000184500 | PROS1 | protein_coding |
| ENSG00000109610 | SOD3 | protein_coding |
| ENSG00000174502 | SLC26A9 | protein_coding |
| ENSG00000091831 | ESR1 | protein_coding |
| ENSG00000088881 | EBF4 | protein_coding |
| ENSG00000172264 | MACROD2 | protein_coding |
| ENSG00000135298 | ADGRB3 | protein_coding |
| ENSG00000101187 | SLCO4A1 | protein_coding |
| ENSG00000230445 | LRRC37A6P | pseudogene |
| ENSG00000138639 | ARHGAP24 | protein_coding |
| ENSG00000066248 | NGEF | protein_coding |
| ENSG00000235961 | PNMA6A | protein_coding |
| ENSG00000145113 | MUC4 | protein_coding |
| ENSG00000181856 | SLC2A4 | protein_coding |
| ENSG00000134775 | FHOD3 | protein_coding |
| ENSG00000173898 | SPTBN2 | protein_coding |
| ENSG00000176387 | HSD11B2 | protein_coding |
| ENSG00000162687 | KCNT2 | protein_coding |
| ENSG00000132639 | SNAP25 | protein_coding |
| ENSG00000197249 | SERPINA1 | protein_coding |
| ENSG00000124920 | MYRF | protein_coding |
| ENSG00000186197 | EDARADD | protein_coding |
| ENSG00000134215 | VAV3 | protein_coding |
| ENSG00000267296 | CEBPA-AS1 | long_non_coding |
| ENSG00000154099 | DNAAF1 | protein_coding |
| ENSG00000198835 | GJC2 | protein_coding |
| ENSG00000280670 | CCDC163 | protein_coding |
| ENSG00000009950 | MLXIPL | protein_coding |
| ENSG00000101230 | ISM1 | protein_coding |
| ENSG00000166415 | WDR72 | protein_coding |
| ENSG00000272899 | AC025594.2 | protein_coding |
| ENSG00000171094 | ALK | protein_coding |
| ENSG00000133475 | GGT2 | protein_coding |
| ENSG00000166016 | ABTB2 | protein_coding |
| ENSG00000143217 | NECTIN4 | protein_coding |
| ENSG00000152137 | HSPB8 | protein_coding |
| ENSG00000237187 | NR2F1-AS1 | long_non_coding |
| ENSG00000231445 | TIMM8AP1 | pseudogene |
| ENSG00000136040 | PLXNC1 | protein_coding |
| ENSG00000119630 | PGF | protein_coding |
| ENSG00000117707 | PROX1 | protein_coding |
| ENSG00000184985 | SORCS2 | protein_coding |
| ENSG00000062282 | DGAT2 | protein_coding |
| ENSG00000102802 | MEDAG | protein_coding |
| ENSG00000162545 | CAMK2N1 | protein_coding |
| ENSG00000102401 | ARMCX3 | protein_coding |
| ENSG00000272168 | CASC15 | long_non_coding |
| ENSG00000172828 | CES3 | protein_coding |
| ENSG00000171766 | GATM | protein_coding |
| ENSG00000156097 | GPR61 | protein_coding |
| ENSG00000009694 | TENM1 | protein_coding |
| ENSG00000213937 | CLDN9 | protein_coding |
| ENSG00000171889 | MIR31HG | long_non_coding |
| ENSG00000182109 | AL365277.1 | long_non_coding |
| ENSG00000110492 | MDK | protein_coding |
| ENSG00000250748 | AC025419.1 | long_non_coding |
| ENSG00000174607 | UGT8 | protein_coding |
| ENSG00000197558 | SSPO | protein_coding |
| ENSG00000168427 | KLHL30 | protein_coding |
| ENSG00000100033 | PRODH | protein_coding |
| ENSG00000101115 | SALL4 | protein_coding |
| ENSG00000189410 | SH2D5 | protein_coding |
| ENSG00000206120 | EGFEM1P | pseudogene |
| ENSG00000173698 | ADGRG2 | protein_coding |
| ENSG00000197635 | DPP4 | protein_coding |
| ENSG00000173267 | SNCG | protein_coding |
| ENSG00000112782 | CLIC5 | protein_coding |
| ENSG00000171303 | KCNK3 | protein_coding |
| ENSG00000183742 | MACC1 | protein_coding |
| ENSG00000256969 | AC007207.2 | long_non_coding |
| ENSG00000141744 | PNMT | protein_coding |
| ENSG00000135318 | NT5E | protein_coding |
| ENSG00000174473 | GALNTL6 | protein_coding |
| ENSG00000157445 | CACNA2D3 | protein_coding |
| ENSG00000164379 | FOXQ1 | protein_coding |
| ENSG00000128040 | SPINK2 | protein_coding |
| ENSG00000134755 | DSC2 | protein_coding |
| ENSG00000274021 | AC024909.3 | long_non_coding |
| ENSG00000163171 | CDC42EP3 | protein_coding |
| ENSG00000196132 | MYT1 | protein_coding |
| ENSG00000223914 | LINC02471 | long_non_coding |
| ENSG00000187867 | PALM3 | protein_coding |
| ENSG00000188910 | GJB3 | protein_coding |
| ENSG00000115221 | ITGB6 | protein_coding |
| ENSG00000203727 | SAMD5 | protein_coding |
| ENSG00000143850 | PLEKHA6 | protein_coding |
| ENSG00000144908 | ALDH1L1 | protein_coding |
| ENSG00000197580 | BCO2 | protein_coding |
| ENSG00000197329 | PELI1 | protein_coding |
| ENSG00000164128 | NPY1R | protein_coding |
| ENSG00000042832 | TG | protein_coding |
| ENSG00000274565 | AC080038.1 | long_non_coding |
| ENSG00000012171 | SEMA3B | protein_coding |
| ENSG00000198915 | RASGEF1A | protein_coding |
| ENSG00000197421 | GGT3P | pseudogene |
| ENSG00000197165 | SULT1A2 | protein_coding |
| ENSG00000237742 | AL365259.1 | long_non_coding |
| ENSG00000132470 | ITGB4 | protein_coding |
| ENSG00000150672 | DLG2 | protein_coding |
| ENSG00000139055 | ERP27 | protein_coding |
| ENSG00000131730 | CKMT2 | protein_coding |
| ENSG00000196083 | IL1RAP | protein_coding |
| ENSG00000173705 | SUSD5 | protein_coding |
| ENSG00000189292 | ALKAL2 | protein_coding |
| ENSG00000183454 | GRIN2A | protein_coding |
| ENSG00000101489 | CELF4 | protein_coding |
| ENSG00000064309 | CDON | protein_coding |
| ENSG00000170153 | RNF150 | protein_coding |
| ENSG00000182389 | CACNB4 | protein_coding |
| ENSG00000152284 | TCF7L1 | protein_coding |
| ENSG00000166793 | YPEL4 | protein_coding |
| ENSG00000125378 | BMP4 | protein_coding |
| ENSG00000163472 | TMEM79 | protein_coding |
| ENSG00000133816 | MICAL2 | protein_coding |
| ENSG00000013619 | MAMLD1 | protein_coding |
| ENSG00000183690 | EFHC2 | protein_coding |
| ENSG00000148795 | CYP17A1 | protein_coding |
| ENSG00000102804 | TSC22D1 | protein_coding |
| ENSG00000162873 | KLHDC8A | protein_coding |
| ENSG00000169715 | MT1E | protein_coding |
| ENSG00000153292 | ADGRF1 | protein_coding |
| ENSG00000141934 | PLPP2 | protein_coding |
| ENSG00000177990 | DPY19L2 | protein_coding |
| ENSG00000211448 | DIO2 | protein_coding |
| ENSG00000184908 | CLCNKB | protein_coding |
| ENSG00000182010 | RTKN2 | protein_coding |
| ENSG00000113594 | LIFR | protein_coding |
| ENSG00000154556 | SORBS2 | protein_coding |
| ENSG00000105376 | ICAM5 | protein_coding |
| ENSG00000171812 | COL8A2 | protein_coding |
| ENSG00000163141 | BNIPL | protein_coding |
| ENSG00000134569 | LRP4 | protein_coding |
| ENSG00000226644 | AL121899.1 | long_non_coding |
| ENSG00000182580 | EPHB3 | protein_coding |
| ENSG00000170382 | LRRN2 | protein_coding |
| ENSG00000133794 | ARNTL | protein_coding |
| ENSG00000151914 | DST | protein_coding |
| ENSG00000188906 | LRRK2 | protein_coding |
| ENSG00000069188 | SDK2 | protein_coding |
| ENSG00000105281 | SLC1A5 | protein_coding |
| ENSG00000259351 | AC015914.1 | long_non_coding |
| ENSG00000128165 | ADM2 | protein_coding |
| ENSG00000145147 | SLIT2 | protein_coding |
| ENSG00000103089 | FA2H | protein_coding |
| ENSG00000076344 | RGS11 | protein_coding |
| ENSG00000103647 | CORO2B | protein_coding |
| ENSG00000153246 | PLA2R1 | protein_coding |
| ENSG00000214491 | SEC14L6 | protein_coding |
| ENSG00000204305 | AGER | protein_coding |
| ENSG00000086991 | NOX4 | protein_coding |
| ENSG00000150687 | PRSS23 | protein_coding |
| ENSG00000174899 | PQLC2L | protein_coding |
| ENSG00000100302 | RASD2 | protein_coding |
| ENSG00000198944 | SOWAHA | protein_coding |
| ENSG00000117122 | MFAP2 | protein_coding |
| ENSG00000143171 | RXRG | protein_coding |
| ENSG00000145623 | OSMR | protein_coding |
| ENSG00000109906 | ZBTB16 | protein_coding |
| ENSG00000125848 | FLRT3 | protein_coding |
| ENSG00000185112 | FAM43A | protein_coding |
| ENSG00000167772 | ANGPTL4 | protein_coding |
| ENSG00000113070 | HBEGF | protein_coding |
| ENSG00000270076 | AF131215.7 | long_non_coding |
| ENSG00000143502 | SUSD4 | protein_coding |
| ENSG00000115616 | SLC9A2 | protein_coding |
| ENSG00000116194 | ANGPTL1 | protein_coding |
| ENSG00000111452 | ADGRD1 | protein_coding |
| ENSG00000105650 | PDE4C | protein_coding |
| ENSG00000172260 | NEGR1 | protein_coding |
| ENSG00000176769 | TCERG1L | protein_coding |
| ENSG00000245848 | CEBPA | protein_coding |
| ENSG00000232973 | CYP1B1-AS1 | long_non_coding |
| ENSG00000197301 | AC090673.1 | long_non_coding |
| ENSG00000188483 | IER5L | protein_coding |
| ENSG00000204540 | PSORS1C1 | protein_coding |
| ENSG00000146197 | SCUBE3 | protein_coding |
| ENSG00000105974 | CAV1 | protein_coding |
| ENSG00000254726 | MEX3A | protein_coding |
| ENSG00000188833 | ENTPD8 | protein_coding |
| ENSG00000105855 | ITGB8 | protein_coding |
| ENSG00000132561 | MATN2 | protein_coding |
| ENSG00000237289 | CKMT1B | protein_coding |
| ENSG00000173083 | HPSE | protein_coding |
| ENSG00000101680 | LAMA1 | protein_coding |
| ENSG00000154736 | ADAMTS5 | protein_coding |
| ENSG00000182795 | C1orf116 | protein_coding |
| ENSG00000187193 | MT1X | protein_coding |
| ENSG00000147883 | CDKN2B | protein_coding |
| ENSG00000142677 | IL22RA1 | protein_coding |
| ENSG00000146072 | TNFRSF21 | protein_coding |
| ENSG00000158292 | GPR153 | protein_coding |
| ENSG00000231107 | LINC01508 | long_non_coding |
| ENSG00000162614 | NEXN | protein_coding |
| ENSG00000124249 | KCNK15 | protein_coding |
| ENSG00000172602 | RND1 | protein_coding |
| ENSG00000204839 | MROH6 | protein_coding |
| ENSG00000184194 | GPR173 | protein_coding |
| ENSG00000267519 | AC020916.1 | long_non_coding |
| ENSG00000176928 | GCNT4 | protein_coding |
| ENSG00000112394 | SLC16A10 | protein_coding |
| ENSG00000103811 | CTSH | protein_coding |
| ENSG00000120885 | CLU | protein_coding |
| ENSG00000134323 | MYCN | protein_coding |
| ENSG00000167183 | PRR15L | protein_coding |
| ENSG00000111319 | SCNN1A | protein_coding |
| ENSG00000180530 | NRIP1 | protein_coding |
| ENSG00000070404 | FSTL3 | protein_coding |
| ENSG00000131435 | PDLIM4 | protein_coding |
| ENSG00000106688 | SLC1A1 | protein_coding |
| ENSG00000005513 | SOX8 | protein_coding |
| ENSG00000164638 | SLC29A4 | protein_coding |
| ENSG00000198003 | CCDC151 | protein_coding |
| ENSG00000166816 | LDHD | protein_coding |
| ENSG00000170324 | FRMPD2 | protein_coding |
| ENSG00000166922 | SCG5 | protein_coding |
| ENSG00000101670 | LIPG | protein_coding |
| ENSG00000203722 | RAET1G | protein_coding |
| ENSG00000115363 | EVA1A | protein_coding |
| ENSG00000155792 | DEPTOR | protein_coding |
| ENSG00000163975 | MELTF | protein_coding |
| ENSG00000168077 | SCARA3 | protein_coding |
| ENSG00000154928 | EPHB1 | protein_coding |
| ENSG00000105427 | CNFN | protein_coding |
| ENSG00000100097 | LGALS1 | protein_coding |
| ENSG00000236740 | AL033384.1 | long_non_coding |
| ENSG00000160352 | ZNF714 | protein_coding |
| ENSG00000119147 | C2orf40 | protein_coding |
| ENSG00000099365 | STX1B | protein_coding |
| ENSG00000141576 | RNF157 | protein_coding |
| ENSG00000060656 | PTPRU | protein_coding |
| ENSG00000137573 | SULF1 | protein_coding |
| ENSG00000163083 | INHBB | protein_coding |
| ENSG00000165996 | HACD1 | protein_coding |
| ENSG00000143845 | ETNK2 | protein_coding |
| ENSG00000132334 | PTPRE | protein_coding |
| ENSG00000139044 | B4GALNT3 | protein_coding |
| ENSG00000203867 | RBM20 | protein_coding |
| ENSG00000160097 | FNDC5 | protein_coding |
| ENSG00000268357 | VN1R81P | pseudogene |
| ENSG00000149575 | SCN2B | protein_coding |
| ENSG00000113231 | PDE8B | protein_coding |
| ENSG00000186007 | LEMD1 | protein_coding |
| ENSG00000164850 | GPER1 | protein_coding |
| ENSG00000276975 | HYDIN2 | pseudogene |
| ENSG00000008277 | ADAM22 | protein_coding |
| ENSG00000131015 | ULBP2 | protein_coding |
| ENSG00000116106 | EPHA4 | protein_coding |
| ENSG00000169047 | IRS1 | protein_coding |
| ENSG00000197956 | S100A6 | protein_coding |
| ENSG00000115963 | RND3 | protein_coding |
| ENSG00000067082 | KLF6 | protein_coding |
| ENSG00000167216 | KATNAL2 | protein_coding |
| ENSG00000112378 | PERP | protein_coding |
| ENSG00000090339 | ICAM1 | protein_coding |
| ENSG00000157111 | TMEM171 | protein_coding |
| ENSG00000120217 | CD274 | protein_coding |
| ENSG00000213390 | ARHGAP19 | protein_coding |
| ENSG00000170500 | LONRF2 | protein_coding |
| ENSG00000127252 | HRASLS | protein_coding |
| ENSG00000076864 | RAP1GAP | protein_coding |
| ENSG00000159166 | LAD1 | protein_coding |
| ENSG00000128849 | CGNL1 | protein_coding |
| ENSG00000270069 | MIR222HG | long_non_coding |
| ENSG00000168280 | KIF5C | protein_coding |
| ENSG00000187642 | PERM1 | protein_coding |
| ENSG00000183784 | C9orf66 | protein_coding |
| ENSG00000177335 | C8orf31 | long_non_coding |
| ENSG00000112182 | BACH2 | protein_coding |
| ENSG00000187185 | AC092118.1 | long_non_coding |
| ENSG00000088836 | SLC4A11 | protein_coding |
| ENSG00000167759 | KLK13 | protein_coding |
| ENSG00000127863 | TNFRSF19 | protein_coding |
| ENSG00000183696 | UPP1 | protein_coding |
| ENSG00000164855 | TMEM184A | protein_coding |
| ENSG00000183379 | SYNDIG1L | protein_coding |
| ENSG00000087085 | ACHE | protein_coding |
| ENSG00000273604 | EPOP | protein_coding |
| ENSG00000127377 | CRYGN | protein_coding |
| ENSG00000196482 | ESRRG | protein_coding |
| ENSG00000151952 | TMEM132D | protein_coding |
| ENSG00000091844 | RGS17 | protein_coding |
| ENSG00000147642 | SYBU | protein_coding |
| ENSG00000256340 | ABCC6P1 | pseudogene |
| ENSG00000172031 | EPHX4 | protein_coding |
| ENSG00000137331 | IER3 | protein_coding |
| ENSG00000157734 | SNX22 | protein_coding |
| ENSG00000183549 | ACSM5 | protein_coding |
| ENSG00000224945 | AL353150.1 | long_non_coding |
| ENSG00000165238 | WNK2 | protein_coding |
| ENSG00000171368 | TPPP | protein_coding |
| ENSG00000126368 | NR1D1 | protein_coding |
| ENSG00000206190 | ATP10A | protein_coding |
| ENSG00000115041 | KCNIP3 | protein_coding |
| ENSG00000047597 | XK | protein_coding |
| ENSG00000148082 | SHC3 | protein_coding |
| ENSG00000176945 | MUC20 | protein_coding |
| ENSG00000125968 | ID1 | protein_coding |
| ENSG00000147100 | SLC16A2 | protein_coding |
| ENSG00000138311 | ZNF365 | protein_coding |
| ENSG00000162105 | SHANK2 | protein_coding |
| ENSG00000159216 | RUNX1 | protein_coding |
| ENSG00000223802 | CERS1 | protein_coding |
| ENSG00000101825 | MXRA5 | protein_coding |
| ENSG00000166349 | RAG1 | protein_coding |
| ENSG00000114279 | FGF12 | protein_coding |
| ENSG00000206077 | ZDHHC11B | protein_coding |
| ENSG00000132563 | REEP2 | protein_coding |
| ENSG00000163884 | KLF15 | protein_coding |
| ENSG00000048740 | CELF2 | protein_coding |
| ENSG00000150893 | FREM2 | protein_coding |
| ENSG00000110076 | NRXN2 | protein_coding |
| ENSG00000182263 | FIGN | protein_coding |
| ENSG00000171714 | ANO5 | protein_coding |
| ENSG00000118898 | PPL | protein_coding |
| ENSG00000164742 | ADCY1 | protein_coding |
| ENSG00000023171 | GRAMD1B | protein_coding |
| ENSG00000138772 | ANXA3 | protein_coding |
| ENSG00000139174 | PRICKLE1 | protein_coding |
| ENSG00000227258 | SMIM2-AS1 | long_non_coding |
| ENSG00000138735 | PDE5A | protein_coding |
| ENSG00000118257 | NRP2 | protein_coding |
| ENSG00000117226 | GBP3 | protein_coding |
| ENSG00000156299 | TIAM1 | protein_coding |
| ENSG00000260711 | AL121839.2 | long_non_coding |
| ENSG00000154229 | PRKCA | protein_coding |
| ENSG00000168490 | PHYHIP | protein_coding |
| ENSG00000134824 | FADS2 | protein_coding |
| ENSG00000059804 | SLC2A3 | protein_coding |
| ENSG00000204860 | FAM201A | long_non_coding |
| ENSG00000144057 | ST6GAL2 | protein_coding |
| ENSG00000260549 | MT1L | pseudogene |
| ENSG00000172771 | EFCAB12 | protein_coding |
| ENSG00000102265 | TIMP1 | protein_coding |
| ENSG00000139211 | AMIGO2 | protein_coding |
| ENSG00000168032 | ENTPD3 | protein_coding |
| ENSG00000126016 | AMOT | protein_coding |
| ENSG00000165548 | TMEM63C | protein_coding |
| ENSG00000105976 | MET | protein_coding |
| ENSG00000064655 | EYA2 | protein_coding |
| ENSG00000065361 | ERBB3 | protein_coding |
| ENSG00000204618 | RNF39 | protein_coding |
| ENSG00000143028 | SYPL2 | protein_coding |
| ENSG00000254343 | AC091563.1 | long_non_coding |
| ENSG00000240859 | AC093627.4 | long_non_coding |
| ENSG00000088756 | ARHGAP28 | protein_coding |
| ENSG00000106100 | NOD1 | protein_coding |
| ENSG00000157470 | FAM81A | protein_coding |
| ENSG00000103044 | HAS3 | protein_coding |
| ENSG00000149599 | DUSP15 | protein_coding |
| ENSG00000106799 | TGFBR1 | protein_coding |
| ENSG00000099864 | PALM | protein_coding |
| ENSG00000115828 | QPCT | protein_coding |
| ENSG00000110042 | DTX4 | protein_coding |
| ENSG00000181019 | NQO1 | protein_coding |
| ENSG00000105270 | CLIP3 | protein_coding |
| ENSG00000181649 | PHLDA2 | protein_coding |
| ENSG00000144802 | NFKBIZ | protein_coding |
| ENSG00000233622 | CYP2T1P | pseudogene |
| ENSG00000164023 | SGMS2 | protein_coding |
| ENSG00000095383 | TBC1D2 | protein_coding |
| ENSG00000124813 | RUNX2 | protein_coding |
| ENSG00000155926 | SLA | protein_coding |
| ENSG00000144824 | PHLDB2 | protein_coding |
| ENSG00000197191 | CYSRT1 | protein_coding |
| ENSG00000267194 | AC002546.1 | long_non_coding |
| ENSG00000185133 | INPP5J | protein_coding |
| ENSG00000006625 | GGCT | protein_coding |
| ENSG00000158246 | FAM46B | protein_coding |
| ENSG00000116661 | FBXO2 | protein_coding |
| ENSG00000105717 | PBX4 | protein_coding |
| ENSG00000163659 | TIPARP | protein_coding |
| ENSG00000176788 | BASP1 | protein_coding |
| ENSG00000111816 | FRK | protein_coding |
| ENSG00000159231 | CBR3 | protein_coding |
| ENSG00000211445 | GPX3 | protein_coding |
| ENSG00000184163 | C1QTNF12 | protein_coding |
| ENSG00000235978 | AC018816.1 | long_non_coding |
| ENSG00000214944 | ARHGEF28 | protein_coding |
| ENSG00000240875 | LINC00886 | long_non_coding |
| ENSG00000057704 | TMCC3 | protein_coding |
| ENSG00000120693 | SMAD9 | protein_coding |
| ENSG00000144730 | IL17RD | protein_coding |
| ENSG00000140545 | MFGE8 | protein_coding |
| ENSG00000180113 | TDRD6 | protein_coding |
| ENSG00000036530 | CYP46A1 | protein_coding |
| ENSG00000247134 | AC090204.1 | long_non_coding |
| ENSG00000244731 | C4A | protein_coding |
| ENSG00000165816 | VWA2 | protein_coding |
| ENSG00000142279 | WTIP | protein_coding |
| ENSG00000275395 | FCGBP | protein_coding |
| ENSG00000132205 | EMILIN2 | protein_coding |
| ENSG00000168255 | POLR2J3 | protein_coding |
| ENSG00000142449 | FBN3 | protein_coding |
| ENSG00000064225 | ST3GAL6 | protein_coding |
| ENSG00000165092 | ALDH1A1 | protein_coding |
| ENSG00000091490 | SEL1L3 | protein_coding |
| ENSG00000017483 | SLC38A5 | protein_coding |
| ENSG00000103742 | IGDCC4 | protein_coding |
| ENSG00000131018 | SYNE1 | protein_coding |
| ENSG00000160932 | LY6E | protein_coding |
| ENSG00000244694 | PTCHD4 | protein_coding |
| ENSG00000005884 | ITGA3 | protein_coding |
| ENSG00000114923 | SLC4A3 | protein_coding |
| ENSG00000270000 | AC005479.3 | long_non_coding |
| ENSG00000165959 | CLMN | protein_coding |
| ENSG00000146267 | FAXC | protein_coding |
| ENSG00000134202 | GSTM3 | protein_coding |
| ENSG00000119938 | PPP1R3C | protein_coding |
| ENSG00000163827 | LRRC2 | protein_coding |
| ENSG00000106546 | AHR | protein_coding |
| ENSG00000116990 | MYCL | protein_coding |
| ENSG00000160678 | S100A1 | protein_coding |
| ENSG00000159307 | SCUBE1 | protein_coding |
| ENSG00000198208 | RPS6KL1 | protein_coding |
| ENSG00000187583 | PLEKHN1 | protein_coding |
| ENSG00000203706 | SERTAD4-AS1 | long_non_coding |
| ENSG00000057657 | PRDM1 | protein_coding |
| ENSG00000087510 | TFAP2C | protein_coding |
| ENSG00000258472 | AC005726.2 | protein_coding |
| ENSG00000162981 | FAM84A | protein_coding |
| ENSG00000167895 | TMC8 | protein_coding |
| ENSG00000256268 | LINC02454 | long_non_coding |
| ENSG00000175040 | CHST2 | protein_coding |
| ENSG00000158457 | TSPAN33 | protein_coding |
| ENSG00000163898 | LIPH | protein_coding |
| ENSG00000181634 | TNFSF15 | protein_coding |
| ENSG00000272079 | AC004233.3 | long_non_coding |
| ENSG00000224389 | C4B | protein_coding |
| ENSG00000164627 | KIF6 | protein_coding |
| ENSG00000218357 | LINC01644 | long_non_coding |
| ENSG00000179133 | C10orf67 | protein_coding |
| ENSG00000213988 | ZNF90 | protein_coding |
| ENSG00000205403 | CFI | protein_coding |
| ENSG00000079101 | CLUL1 | protein_coding |
| ENSG00000167081 | PBX3 | protein_coding |
| ENSG00000245571 | AP001258.1 | long_non_coding |
| ENSG00000261488 | AC128688.2 | long_non_coding |
| ENSG00000241749 | RPSAP52 | pseudogene |
| ENSG00000128342 | LIF | protein_coding |
| ENSG00000180155 | LYNX1 | protein_coding |
| ENSG00000179862 | CITED4 | protein_coding |
| ENSG00000215481 | BCRP3 | long_non_coding |
| ENSG00000231991 | ANXA2P2 | pseudogene |
| ENSG00000036448 | MYOM2 | protein_coding |
| ENSG00000036672 | USP2 | protein_coding |
| ENSG00000113657 | DPYSL3 | protein_coding |
| ENSG00000056998 | GYG2 | protein_coding |
| ENSG00000111254 | AKAP3 | protein_coding |
| ENSG00000111181 | SLC6A12 | protein_coding |
| ENSG00000243836 | WDR86-AS1 | long_non_coding |
| ENSG00000250479 | CHCHD10 | protein_coding |
| ENSG00000272970 | AC107294.2 | long_non_coding |
| ENSG00000188001 | TPRG1 | protein_coding |
| ENSG00000180071 | ANKRD18A | protein_coding |
| ENSG00000215218 | UBE2QL1 | protein_coding |
| ENSG00000005243 | COPZ2 | protein_coding |
| ENSG00000161714 | PLCD3 | protein_coding |
| ENSG00000130701 | RBBP8NL | protein_coding |
| ENSG00000203485 | INF2 | protein_coding |
| ENSG00000205542 | TMSB4X | protein_coding |
| ENSG00000005001 | PRSS22 | protein_coding |
| ENSG00000163406 | SLC15A2 | protein_coding |
| ENSG00000163485 | ADORA1 | protein_coding |
| ENSG00000158352 | SHROOM4 | protein_coding |
| ENSG00000170379 | TCAF2 | protein_coding |
| ENSG00000164794 | KCNV1 | protein_coding |
| ENSG00000225742 | LINC02036 | long_non_coding |
| ENSG00000134107 | BHLHE40 | protein_coding |
| ENSG00000138670 | RASGEF1B | protein_coding |
| ENSG00000184305 | CCSER1 | protein_coding |
| ENSG00000147408 | CSGALNACT1 | protein_coding |
| ENSG00000215912 | TTC34 | protein_coding |
| ENSG00000011028 | MRC2 | protein_coding |
| ENSG00000155324 | GRAMD2B | protein_coding |
| ENSG00000162444 | RBP7 | protein_coding |
| ENSG00000138685 | FGF2 | protein_coding |
| ENSG00000147852 | VLDLR | protein_coding |
| ENSG00000111052 | LIN7A | protein_coding |
| ENSG00000239521 | GATS | protein_coding |
| ENSG00000165995 | CACNB2 | protein_coding |
| ENSG00000164078 | MST1R | protein_coding |
| ENSG00000224307 | AL161785.1 | long_non_coding |
| ENSG00000170011 | MYRIP | protein_coding |
| ENSG00000232653 | GOLGA8N | protein_coding |
| ENSG00000137941 | TTLL7 | protein_coding |
| ENSG00000196502 | SULT1A1 | protein_coding |
| ENSG00000133026 | MYH10 | protein_coding |
| ENSG00000213985 | AC078899.1 | pseudogene |
| ENSG00000155158 | TTC39B | protein_coding |
| ENSG00000146021 | KLHL3 | protein_coding |
| ENSG00000154655 | L3MBTL4 | protein_coding |
| ENSG00000124145 | SDC4 | protein_coding |
| ENSG00000124766 | SOX4 | protein_coding |
| ENSG00000166450 | PRTG | protein_coding |
| ENSG00000236975 | AL137793.1 | long_non_coding |
| ENSG00000198468 | FLVCR1-AS1 | long_non_coding |
| ENSG00000198691 | ABCA4 | protein_coding |
| ENSG00000108515 | ENO3 | protein_coding |
| ENSG00000142227 | EMP3 | protein_coding |
| ENSG00000196420 | S100A5 | protein_coding |
| ENSG00000123119 | NECAB1 | protein_coding |
| ENSG00000010310 | GIPR | protein_coding |
| ENSG00000173599 | PC | protein_coding |
| ENSG00000267365 | KCNJ2-AS1 | long_non_coding |
| ENSG00000167617 | CDC42EP5 | protein_coding |
| ENSG00000243710 | CFAP57 | protein_coding |
| ENSG00000185739 | SRL | protein_coding |
| ENSG00000239887 | C1orf226 | protein_coding |
| ENSG00000136048 | DRAM1 | protein_coding |
| ENSG00000136861 | CDK5RAP2 | protein_coding |
| ENSG00000198948 | MFAP3L | protein_coding |
| ENSG00000111912 | NCOA7 | protein_coding |
| ENSG00000263961 | C1orf186 | protein_coding |
| ENSG00000100003 | SEC14L2 | protein_coding |
| ENSG00000141738 | GRB7 | protein_coding |
| ENSG00000258311 | AC009779.3 | protein_coding |
| ENSG00000116260 | QSOX1 | protein_coding |
| ENSG00000186854 | TRABD2A | protein_coding |
| ENSG00000123836 | PFKFB2 | protein_coding |
| ENSG00000250305 | KIAA1456 | protein_coding |
| ENSG00000258999 | AL136040.1 | long_non_coding |
| ENSG00000006327 | TNFRSF12A | protein_coding |
| ENSG00000163874 | ZC3H12A | protein_coding |
| ENSG00000186205 | MARC1 | protein_coding |
| ENSG00000269899 | AC025857.2 | long_non_coding |
| ENSG00000109063 | MYH3 | protein_coding |
| ENSG00000144810 | COL8A1 | protein_coding |
| ENSG00000170745 | KCNS3 | protein_coding |
| ENSG00000122176 | FMOD | protein_coding |
| ENSG00000170439 | METTL7B | protein_coding |
| ENSG00000244968 | LIFR-AS1 | long_non_coding |
| ENSG00000064195 | DLX3 | protein_coding |
| ENSG00000167693 | NXN | protein_coding |
| ENSG00000255366 | AC120036.5 | long_non_coding |
| ENSG00000109738 | GLRB | protein_coding |
| ENSG00000198300 | PEG3 | protein_coding |
| ENSG00000168646 | AXIN2 | protein_coding |
| ENSG00000133116 | KL | protein_coding |
| ENSG00000132518 | GUCY2D | protein_coding |
| ENSG00000176170 | SPHK1 | protein_coding |
| ENSG00000128487 | SPECC1 | protein_coding |
| ENSG00000227825 | SLC9A7P1 | pseudogene |
| ENSG00000233593 | AL590094.1 | long_non_coding |
| ENSG00000256229 | ZNF486 | protein_coding |
| ENSG00000136111 | TBC1D4 | protein_coding |
| ENSG00000020577 | SAMD4A | protein_coding |
| ENSG00000165617 | DACT1 | protein_coding |
| ENSG00000184588 | PDE4B | protein_coding |
| ENSG00000226380 | AC016831.1 | long_non_coding |
| ENSG00000100321 | SYNGR1 | protein_coding |
| ENSG00000267259 | AC061975.7 | long_non_coding |
| ENSG00000105559 | PLEKHA4 | protein_coding |
| ENSG00000136327 | NKX2-8 | protein_coding |
| ENSG00000257261 | AC008014.1 | long_non_coding |
| ENSG00000177707 | NECTIN3 | protein_coding |
| ENSG00000180914 | OXTR | protein_coding |
| ENSG00000165434 | PGM2L1 | protein_coding |
| ENSG00000172817 | CYP7B1 | protein_coding |
| ENSG00000112715 | VEGFA | protein_coding |
| ENSG00000118620 | ZNF430 | protein_coding |
| ENSG00000174514 | MFSD4A | protein_coding |
| ENSG00000163347 | CLDN1 | protein_coding |
| ENSG00000162576 | MXRA8 | protein_coding |
| ENSG00000108932 | SLC16A6 | protein_coding |
| ENSG00000162817 | C1orf115 | protein_coding |
| ENSG00000279118 | AC093535.2 | TEC |
| ENSG00000137309 | HMGA1 | protein_coding |
| ENSG00000235997 | LINC01936 | long_non_coding |
| ENSG00000171791 | BCL2 | protein_coding |
| ENSG00000204991 | SPIRE2 | protein_coding |
| ENSG00000277013 | AC008556.1 | long_non_coding |
| ENSG00000170214 | ADRA1B | protein_coding |
| ENSG00000185022 | MAFF | protein_coding |
| ENSG00000183248 | PRR36 | protein_coding |
| ENSG00000251615 | AC104825.2 | long_non_coding |
| ENSG00000077092 | RARB | protein_coding |
| ENSG00000198221 | AFDN-AS1 | long_non_coding |
| ENSG00000164442 | CITED2 | protein_coding |
| ENSG00000184584 | TMEM173 | protein_coding |
| ENSG00000163590 | PPM1L | protein_coding |
| ENSG00000149260 | CAPN5 | protein_coding |
| ENSG00000197927 | C2orf27A | pseudogene |
| ENSG00000148180 | GSN | protein_coding |
| ENSG00000196218 | RYR1 | protein_coding |
| ENSG00000062038 | CDH3 | protein_coding |
| ENSG00000108830 | RND2 | protein_coding |
| ENSG00000112964 | GHR | protein_coding |
| ENSG00000173890 | GPR160 | protein_coding |
| ENSG00000182718 | ANXA2 | protein_coding |
| ENSG00000164574 | GALNT10 | protein_coding |
| ENSG00000214595 | EML6 | protein_coding |
| ENSG00000068650 | ATP11A | protein_coding |
| ENSG00000142621 | FHAD1 | protein_coding |
| ENSG00000171811 | CFAP46 | protein_coding |
| ENSG00000102996 | MMP15 | protein_coding |
| ENSG00000196155 | PLEKHG4 | protein_coding |
| ENSG00000162496 | DHRS3 | protein_coding |
| ENSG00000258844 | AL162511.1 | long_non_coding |
| ENSG00000136826 | KLF4 | protein_coding |
| ENSG00000105707 | HPN | protein_coding |
| ENSG00000085552 | IGSF9 | protein_coding |
| ENSG00000099256 | PRTFDC1 | protein_coding |
| ENSG00000204103 | MAFB | protein_coding |
| ENSG00000140675 | SLC5A2 | protein_coding |
| ENSG00000197748 | CFAP43 | protein_coding |
| ENSG00000129646 | QRICH2 | protein_coding |
| ENSG00000116717 | GADD45A | protein_coding |
| ENSG00000173535 | TNFRSF10C | protein_coding |
| ENSG00000100379 | KCTD17 | protein_coding |
| ENSG00000105971 | CAV2 | protein_coding |
| ENSG00000123095 | BHLHE41 | protein_coding |
| ENSG00000260400 | AL513534.1 | long_non_coding |
| ENSG00000078018 | MAP2 | protein_coding |
| ENSG00000162595 | DIRAS3 | protein_coding |
| ENSG00000117318 | ID3 | protein_coding |
| ENSG00000149150 | SLC43A1 | protein_coding |
| ENSG00000026103 | FAS | protein_coding |
| ENSG00000162643 | WDR63 | protein_coding |
| ENSG00000065833 | ME1 | protein_coding |
| ENSG00000185437 | SH3BGR | protein_coding |
| ENSG00000137501 | SYTL2 | protein_coding |
| ENSG00000143333 | RGS16 | protein_coding |
| ENSG00000075426 | FOSL2 | protein_coding |
| ENSG00000197442 | MAP3K5 | protein_coding |
| ENSG00000186868 | MAPT | protein_coding |
| ENSG00000170390 | DCLK2 | protein_coding |
| ENSG00000131981 | LGALS3 | protein_coding |
| ENSG00000166165 | CKB | protein_coding |
| ENSG00000119927 | GPAM | protein_coding |
| ENSG00000174791 | RIN1 | protein_coding |
| ENSG00000115290 | GRB14 | protein_coding |
| ENSG00000134531 | EMP1 | protein_coding |
| ENSG00000159239 | AC005041.1 | protein_coding |
| ENSG00000144476 | ACKR3 | protein_coding |
| ENSG00000250608 | AC010210.1 | long_non_coding |
| ENSG00000113448 | PDE4D | protein_coding |
| ENSG00000185950 | IRS2 | protein_coding |
| ENSG00000246640 | PICART1 | long_non_coding |
| ENSG00000142959 | BEST4 | protein_coding |
| ENSG00000221890 | NPTXR | protein_coding |
| ENSG00000196705 | ZNF431 | protein_coding |
| ENSG00000172818 | OVOL1 | protein_coding |
| ENSG00000168140 | VASN | protein_coding |
| ENSG00000188549 | C15orf52 | protein_coding |
| ENSG00000170271 | FAXDC2 | protein_coding |
| ENSG00000167996 | FTH1 | protein_coding |
| ENSG00000135205 | CCDC146 | protein_coding |
| ENSG00000197020 | ZNF100 | protein_coding |
| ENSG00000185046 | ANKS1B | protein_coding |
| ENSG00000108984 | MAP2K6 | protein_coding |
| ENSG00000172915 | NBEA | protein_coding |
| ENSG00000187699 | C2orf88 | protein_coding |
| ENSG00000006534 | ALDH3B1 | protein_coding |
| ENSG00000179023 | KLHDC7A | protein_coding |
| ENSG00000178162 | FAR2P2 | pseudogene |
| ENSG00000003987 | MTMR7 | protein_coding |
| ENSG00000039560 | RAI14 | protein_coding |
| ENSG00000160191 | PDE9A | protein_coding |
| ENSG00000224020 | MIR181A2HG | long_non_coding |
| ENSG00000196711 | ALKAL1 | protein_coding |
| ENSG00000136014 | USP44 | protein_coding |
| ENSG00000167103 | PIP5KL1 | protein_coding |
| ENSG00000260992 | DOCK9-AS2 | long_non_coding |
| ENSG00000196381 | ZNF781 | protein_coding |
| ENSG00000162433 | AK4 | protein_coding |
| ENSG00000277363 | SRCIN1 | protein_coding |
| ENSG00000233198 | RNF224 | protein_coding |
| ENSG00000035664 | DAPK2 | protein_coding |
| ENSG00000105755 | ETHE1 | protein_coding |
| ENSG00000204282 | TNRC6C-AS1 | long_non_coding |
| ENSG00000163235 | TGFA | protein_coding |
| ENSG00000172738 | TMEM217 | protein_coding |
| ENSG00000163075 | CFAP221 | protein_coding |
| ENSG00000156463 | SH3RF2 | protein_coding |
| ENSG00000139318 | DUSP6 | protein_coding |
| ENSG00000006042 | TMEM98 | protein_coding |
| ENSG00000130751 | NPAS1 | protein_coding |
| ENSG00000132970 | WASF3 | protein_coding |
| ENSG00000185332 | TMEM105 | protein_coding |
| ENSG00000237424 | FOXD2-AS1 | long_non_coding |
| ENSG00000102098 | SCML2 | protein_coding |
| ENSG00000088387 | DOCK9 | protein_coding |
| ENSG00000081059 | TCF7 | protein_coding |
| ENSG00000246985 | SOCS2-AS1 | long_non_coding |
| ENSG00000273382 | AL356488.3 | long_non_coding |
| ENSG00000131773 | KHDRBS3 | protein_coding |
| ENSG00000166402 | TUB | protein_coding |
| ENSG00000114315 | HES1 | protein_coding |
| ENSG00000162591 | MEGF6 | protein_coding |
| ENSG00000072954 | TMEM38A | protein_coding |
| ENSG00000154122 | ANKH | protein_coding |
| ENSG00000258818 | RNASE4 | protein_coding |
| ENSG00000108960 | MMD | protein_coding |
| ENSG00000275183 | LENG9 | protein_coding |
| ENSG00000111907 | TPD52L1 | protein_coding |
| ENSG00000120055 | C10orf95 | protein_coding |
| ENSG00000260920 | AL031985.3 | long_non_coding |
| ENSG00000168491 | CCDC110 | protein_coding |
| ENSG00000188153 | COL4A5 | protein_coding |
| ENSG00000196139 | AKR1C3 | protein_coding |
| ENSG00000014914 | MTMR11 | protein_coding |
| ENSG00000111275 | ALDH2 | protein_coding |
| ENSG00000116678 | LEPR | protein_coding |
| ENSG00000259583 | AC015712.2 | long_non_coding |
| ENSG00000128266 | GNAZ | protein_coding |
| ENSG00000222041 | CYTOR | long_non_coding |
| ENSG00000250899 | AC125807.2 | long_non_coding |
| ENSG00000260196 | AC124798.1 | long_non_coding |
| ENSG00000107485 | GATA3 | protein_coding |
| ENSG00000116985 | BMP8B | protein_coding |
| ENSG00000170006 | TMEM154 | protein_coding |
| ENSG00000136404 | TM6SF1 | protein_coding |
| ENSG00000144152 | FBLN7 | protein_coding |
| ENSG00000116771 | AGMAT | protein_coding |
| ENSG00000076382 | SPAG5 | protein_coding |
| ENSG00000233901 | LINC01503 | long_non_coding |
| ENSG00000205710 | C17orf107 | protein_coding |
| ENSG00000159173 | TNNI1 | protein_coding |
| ENSG00000104369 | JPH1 | protein_coding |
| ENSG00000214274 | ANG | protein_coding |
| ENSG00000105711 | SCN1B | protein_coding |
| ENSG00000177283 | FZD8 | protein_coding |
| ENSG00000183840 | GPR39 | protein_coding |
| ENSG00000170525 | PFKFB3 | protein_coding |
| ENSG00000089327 | FXYD5 | protein_coding |
| ENSG00000228559 | AL033519.3 | long_non_coding |
| ENSG00000128805 | ARHGAP22 | protein_coding |
| ENSG00000170629 | DPY19L2P2 | pseudogene |
| ENSG00000162931 | TRIM17 | protein_coding |
| ENSG00000134297 | PLEKHA8P1 | pseudogene |
| ENSG00000120549 | KIAA1217 | protein_coding |
| ENSG00000138678 | GPAT3 | protein_coding |
| ENSG00000225986 | UBXN10-AS1 | long_non_coding |
| ENSG00000184925 | LCN12 | protein_coding |
| ENSG00000158321 | AUTS2 | protein_coding |
| ENSG00000250343 | STK32A-AS1 | long_non_coding |
| ENSG00000079156 | OSBPL6 | protein_coding |
| ENSG00000186231 | KLHL32 | protein_coding |
| ENSG00000144136 | SLC20A1 | protein_coding |
| ENSG00000184164 | CRELD2 | protein_coding |
| ENSG00000205089 | CCNI2 | protein_coding |
| ENSG00000151692 | RNF144A | protein_coding |
| ENSG00000140057 | AK7 | protein_coding |
| ENSG00000234465 | PINLYP | protein_coding |
| ENSG00000226124 | FTCDNL1 | protein_coding |
| ENSG00000205791 | LOH12CR2 | long_non_coding |
| ENSG00000111012 | CYP27B1 | protein_coding |
| ENSG00000254087 | LYN | protein_coding |
| ENSG00000187801 | ZFP69B | protein_coding |
| ENSG00000169750 | RAC3 | protein_coding |
| ENSG00000072133 | RPS6KA6 | protein_coding |
| ENSG00000196924 | FLNA | protein_coding |
| ENSG00000104205 | SGK3 | protein_coding |
| ENSG00000173852 | DPY19L1 | protein_coding |
| ENSG00000111110 | PPM1H | protein_coding |
| ENSG00000143797 | MBOAT2 | protein_coding |
| ENSG00000260572 | AC069224.1 | long_non_coding |
| ENSG00000123096 | SSPN | protein_coding |
| ENSG00000054690 | PLEKHH1 | protein_coding |
| ENSG00000186594 | MIR22HG | long_non_coding |
| ENSG00000149218 | ENDOD1 | protein_coding |
| ENSG00000138185 | ENTPD1 | protein_coding |
| ENSG00000232533 | AC093673.1 | long_non_coding |
| ENSG00000250091 | DNAH10OS | protein_coding |
| ENSG00000092445 | TYRO3 | protein_coding |
| ENSG00000115598 | IL1RL2 | protein_coding |
| ENSG00000268119 | AC010615.2 | long_non_coding |
| ENSG00000149428 | HYOU1 | protein_coding |
| ENSG00000184574 | LPAR5 | protein_coding |
| ENSG00000153237 | CCDC148 | protein_coding |
| ENSG00000073060 | SCARB1 | protein_coding |
| ENSG00000131389 | SLC6A6 | protein_coding |
| ENSG00000147174 | GCNA | protein_coding |
| ENSG00000186352 | ANKRD37 | protein_coding |
| ENSG00000232453 | AC105277.1 | long_non_coding |
| ENSG00000248727 | LINC01948 | long_non_coding |
| ENSG00000124374 | PAIP2B | protein_coding |
| ENSG00000163191 | S100A11 | protein_coding |
| ENSG00000163328 | GPR155 | protein_coding |
| ENSG00000106069 | CHN2 | protein_coding |
| ENSG00000169302 | STK32A | protein_coding |
| ENSG00000250486 | FAM218A | protein_coding |
| ENSG00000141540 | TTYH2 | protein_coding |
| ENSG00000120262 | CCDC170 | protein_coding |
| ENSG00000143147 | GPR161 | protein_coding |
| ENSG00000143416 | SELENBP1 | protein_coding |
| ENSG00000155254 | MARVELD1 | protein_coding |
| ENSG00000058085 | LAMC2 | protein_coding |
| ENSG00000131899 | LLGL1 | protein_coding |
| ENSG00000130962 | PRRG1 | protein_coding |
| ENSG00000233237 | LINC00472 | long_non_coding |
| ENSG00000140931 | CMTM3 | protein_coding |
| ENSG00000111962 | UST | protein_coding |
| ENSG00000123384 | LRP1 | protein_coding |
| ENSG00000241684 | ADAMTS9-AS2 | long_non_coding |
| ENSG00000125744 | RTN2 | protein_coding |
| ENSG00000040608 | RTN4R | protein_coding |
| ENSG00000140527 | WDR93 | protein_coding |
| ENSG00000156140 | ADAMTS3 | protein_coding |
| ENSG00000197208 | SLC22A4 | protein_coding |
| ENSG00000118922 | KLF12 | protein_coding |
| ENSG00000101460 | MAP1LC3A | protein_coding |
| ENSG00000135046 | ANXA1 | protein_coding |
| ENSG00000175591 | P2RY2 | protein_coding |
| ENSG00000177706 | FAM20C | protein_coding |
| ENSG00000114251 | WNT5A | protein_coding |
| ENSG00000108846 | ABCC3 | protein_coding |
| ENSG00000182013 | PNMA8A | protein_coding |
| ENSG00000149564 | ESAM | protein_coding |
| ENSG00000155252 | PI4K2A | protein_coding |
| ENSG00000105327 | BBC3 | protein_coding |
| ENSG00000262691 | AC040160.1 | long_non_coding |
| ENSG00000184545 | DUSP8 | protein_coding |
| ENSG00000106780 | MEGF9 | protein_coding |
| ENSG00000257556 | LINC02298 | long_non_coding |
| ENSG00000149573 | MPZL2 | protein_coding |
| ENSG00000203797 | DDO | protein_coding |
| ENSG00000116133 | DHCR24 | protein_coding |
| ENSG00000182050 | MGAT4C | protein_coding |
| ENSG00000089847 | ANKRD24 | protein_coding |
| ENSG00000100196 | KDELR3 | protein_coding |
| ENSG00000198729 | PPP1R14C | protein_coding |
| ENSG00000138400 | MDH1B | protein_coding |
| ENSG00000130988 | RGN | protein_coding |
| ENSG00000171208 | NETO2 | protein_coding |
| ENSG00000172201 | ID4 | protein_coding |
| ENSG00000133661 | SFTPD | protein_coding |
| ENSG00000140470 | ADAMTS17 | protein_coding |
| ENSG00000119655 | NPC2 | protein_coding |
| ENSG00000112773 | FAM46A | protein_coding |
| ENSG00000183049 | CAMK1D | protein_coding |
| ENSG00000127249 | ATP13A4 | protein_coding |
| ENSG00000276007 | AC079414.3 | long_non_coding |
| ENSG00000146038 | DCDC2 | protein_coding |
| ENSG00000238278 | ALG1L6P | pseudogene |
| ENSG00000156011 | PSD3 | protein_coding |
| ENSG00000244479 | OR2A1-AS1 | long_non_coding |
| ENSG00000142669 | SH3BGRL3 | protein_coding |
| ENSG00000146648 | EGFR | protein_coding |
| ENSG00000175643 | RMI2 | protein_coding |
| ENSG00000044574 | HSPA5 | protein_coding |
| ENSG00000120162 | MOB3B | protein_coding |
| ENSG00000175906 | ARL4D | protein_coding |
| ENSG00000261438 | AL157394.1 | long_non_coding |
| ENSG00000107438 | PDLIM1 | protein_coding |
| ENSG00000101400 | SNTA1 | protein_coding |
| ENSG00000198960 | ARMCX6 | protein_coding |
| ENSG00000169122 | FAM110B | protein_coding |
| ENSG00000034510 | TMSB10 | protein_coding |
| ENSG00000107731 | UNC5B | protein_coding |
| ENSG00000100767 | PAPLN | protein_coding |
| ENSG00000272695 | GAS6-AS2 | long_non_coding |
| ENSG00000152778 | IFIT5 | protein_coding |
| ENSG00000087842 | PIR | protein_coding |
| ENSG00000067191 | CACNB1 | protein_coding |
| ENSG00000197959 | DNM3 | protein_coding |
| ENSG00000197980 | LEKR1 | protein_coding |
| ENSG00000250073 | AP000866.2 | long_non_coding |
| ENSG00000119943 | PYROXD2 | protein_coding |
| ENSG00000198964 | SGMS1 | protein_coding |
| ENSG00000118997 | DNAH7 | protein_coding |
| ENSG00000091129 | NRCAM | protein_coding |
| ENSG00000110328 | GALNT18 | protein_coding |
| ENSG00000117643 | MAN1C1 | protein_coding |
| ENSG00000177119 | ANO6 | protein_coding |
| ENSG00000214708 | AC116407.1 | long_non_coding |
| ENSG00000181035 | SLC25A42 | protein_coding |
| ENSG00000106571 | GLI3 | protein_coding |
| ENSG00000117791 | MARC2 | protein_coding |
| ENSG00000281406 | BLACAT1 | long_non_coding |
| ENSG00000178814 | OPLAH | protein_coding |
| ENSG00000171914 | TLN2 | protein_coding |
| ENSG00000224596 | ZMIZ1-AS1 | long_non_coding |
| ENSG00000263400 | TMEM220-AS1 | long_non_coding |
| ENSG00000137460 | FHDC1 | protein_coding |
| ENSG00000136237 | RAPGEF5 | protein_coding |
| ENSG00000145050 | MANF | protein_coding |
| ENSG00000151229 | SLC2A13 | protein_coding |
| ENSG00000076555 | ACACB | protein_coding |
| ENSG00000143669 | LYST | protein_coding |
| ENSG00000106789 | CORO2A | protein_coding |
| ENSG00000171552 | BCL2L1 | protein_coding |
| ENSG00000152782 | PANK1 | protein_coding |
| ENSG00000137709 | POU2F3 | protein_coding |
| ENSG00000099625 | CBARP | protein_coding |
| ENSG00000135547 | HEY2 | protein_coding |
| ENSG00000144668 | ITGA9 | protein_coding |
| ENSG00000151929 | BAG3 | protein_coding |
| ENSG00000188766 | SPRED3 | protein_coding |
| ENSG00000153064 | BANK1 | protein_coding |
| ENSG00000171612 | SLC25A33 | protein_coding |
| ENSG00000260577 | AC126773.2 | long_non_coding |
| ENSG00000162512 | SDC3 | protein_coding |
| ENSG00000217555 | CKLF | protein_coding |
| ENSG00000187260 | WDR86 | protein_coding |
| ENSG00000142875 | PRKACB | protein_coding |
| ENSG00000182771 | GRID1 | protein_coding |
| ENSG00000101871 | MID1 | protein_coding |
| ENSG00000230074 | AL162231.2 | long_non_coding |
| ENSG00000095539 | SEMA4G | protein_coding |
| ENSG00000166145 | SPINT1 | protein_coding |
| ENSG00000128283 | CDC42EP1 | protein_coding |
| ENSG00000167552 | TUBA1A | protein_coding |
| ENSG00000233834 | AC005083.1 | long_non_coding |
| ENSG00000135749 | PCNX2 | protein_coding |
| ENSG00000119681 | LTBP2 | protein_coding |
| ENSG00000171130 | ATP6V0E2 | protein_coding |
| ENSG00000101224 | CDC25B | protein_coding |
| ENSG00000075275 | CELSR1 | protein_coding |
| ENSG00000151849 | CENPJ | protein_coding |
| ENSG00000249592 | AC139887.2 | long_non_coding |
| ENSG00000111644 | ACRBP | protein_coding |
| ENSG00000275832 | ARHGAP23 | protein_coding |
| ENSG00000102743 | SLC25A15 | protein_coding |
| ENSG00000137266 | SLC22A23 | protein_coding |
| ENSG00000186472 | PCLO | protein_coding |
| ENSG00000102699 | PARP4 | protein_coding |
| ENSG00000171159 | C9orf16 | protein_coding |
| ENSG00000175567 | UCP2 | protein_coding |
| ENSG00000154217 | PITPNC1 | protein_coding |
| ENSG00000127585 | FBXL16 | protein_coding |
| ENSG00000112290 | WASF1 | protein_coding |
| ENSG00000175505 | CLCF1 | protein_coding |
| ENSG00000173848 | NET1 | protein_coding |
| ENSG00000235609 | AF127577.4 | long_non_coding |
| ENSG00000170522 | ELOVL6 | protein_coding |
| ENSG00000108375 | RNF43 | protein_coding |
| ENSG00000109586 | GALNT7 | protein_coding |
| ENSG00000249602 | AL589765.4 | long_non_coding |
| ENSG00000177191 | B3GNT8 | protein_coding |
| ENSG00000187824 | TMEM220 | protein_coding |
| ENSG00000120875 | DUSP4 | protein_coding |
| ENSG00000143375 | CGN | protein_coding |
| ENSG00000135424 | ITGA7 | protein_coding |
| ENSG00000204934 | ATP6V0E2-AS1 | long_non_coding |
| ENSG00000171621 | SPSB1 | protein_coding |
| ENSG00000196562 | SULF2 | protein_coding |
| ENSG00000128536 | CDHR3 | protein_coding |
| ENSG00000189143 | CLDN4 | protein_coding |
| ENSG00000142611 | PRDM16 | protein_coding |
| ENSG00000184489 | PTP4A3 | protein_coding |
| ENSG00000082146 | STRADB | protein_coding |
| ENSG00000213853 | EMP2 | protein_coding |
| ENSG00000087460 | GNAS | protein_coding |
| ENSG00000206567 | AC022007.1 | long_non_coding |
| ENSG00000021300 | PLEKHB1 | protein_coding |
| ENSG00000139173 | TMEM117 | protein_coding |
| ENSG00000117308 | GALE | protein_coding |
| ENSG00000102003 | SYP | protein_coding |
| ENSG00000184005 | ST6GALNAC3 | protein_coding |
| ENSG00000104635 | SLC39A14 | protein_coding |
| ENSG00000158470 | B4GALT5 | protein_coding |
| ENSG00000167315 | ACAA2 | protein_coding |
| ENSG00000115419 | GLS | protein_coding |
| ENSG00000229689 | AC009237.3 | pseudogene |
| ENSG00000104689 | TNFRSF10A | protein_coding |
| ENSG00000185650 | ZFP36L1 | protein_coding |
| ENSG00000145779 | TNFAIP8 | protein_coding |
| ENSG00000269609 | RPARP-AS1 | long_non_coding |
| ENSG00000151491 | EPS8 | protein_coding |
| ENSG00000261754 | AC008555.1 | long_non_coding |
| ENSG00000127955 | GNAI1 | protein_coding |
| ENSG00000237310 | GS1-124K5.4 | long_non_coding |
| ENSG00000138606 | SHF | protein_coding |
| ENSG00000135272 | MDFIC | protein_coding |
| ENSG00000135338 | LCA5 | protein_coding |
| ENSG00000188277 | C15orf62 | protein_coding |
| ENSG00000141391 | PRELID3A | protein_coding |
| ENSG00000140284 | SLC27A2 | protein_coding |
| ENSG00000167601 | AXL | protein_coding |
| ENSG00000147872 | PLIN2 | protein_coding |
| ENSG00000163040 | CCDC74A | protein_coding |
| ENSG00000134070 | IRAK2 | protein_coding |
| ENSG00000173846 | PLK3 | protein_coding |
| ENSG00000157827 | FMNL2 | protein_coding |
| ENSG00000174567 | GOLT1A | protein_coding |
| ENSG00000125864 | BFSP1 | protein_coding |
| ENSG00000106785 | TRIM14 | protein_coding |
| ENSG00000164251 | F2RL1 | protein_coding |
| ENSG00000172986 | GXYLT2 | protein_coding |
| ENSG00000133121 | STARD13 | protein_coding |
| ENSG00000107537 | PHYH | protein_coding |
| ENSG00000142627 | EPHA2 | protein_coding |
| ENSG00000227199 | ST7-AS1 | long_non_coding |
| ENSG00000197837 | HIST4H4 | protein_coding |
| ENSG00000164741 | DLC1 | protein_coding |
| ENSG00000013364 | MVP | protein_coding |
| ENSG00000256235 | SMIM3 | protein_coding |
| ENSG00000171227 | TMEM37 | protein_coding |
| ENSG00000259248 | USP3-AS1 | long_non_coding |
| ENSG00000143324 | XPR1 | protein_coding |
| ENSG00000102007 | PLP2 | protein_coding |
| ENSG00000173638 | SLC19A1 | protein_coding |
| ENSG00000255085 | AF186192.2 | pseudogene |
| ENSG00000140905 | GCSH | protein_coding |
| ENSG00000066629 | EML1 | protein_coding |
| ENSG00000182985 | CADM1 | protein_coding |
| ENSG00000259291 | ZNF710-AS1 | long_non_coding |
| ENSG00000244026 | FAM86DP | pseudogene |
| ENSG00000124243 | BCAS4 | protein_coding |
| ENSG00000165215 | CLDN3 | protein_coding |
| ENSG00000015475 | BID | protein_coding |
| ENSG00000103599 | IQCH | protein_coding |
| ENSG00000108510 | MED13 | protein_coding |
| ENSG00000213073 | AL353625.1 | pseudogene |
| ENSG00000102057 | KCND1 | protein_coding |
| ENSG00000162999 | DUSP19 | protein_coding |
| ENSG00000109339 | MAPK10 | protein_coding |
| ENSG00000168672 | FAM84B | protein_coding |
| ENSG00000279133 | AC018628.1 | TEC |
| ENSG00000137124 | ALDH1B1 | protein_coding |
| ENSG00000174307 | PHLDA3 | protein_coding |
| ENSG00000111666 | CHPT1 | protein_coding |
| ENSG00000110660 | SLC35F2 | protein_coding |
| ENSG00000181322 | NME9 | protein_coding |
| ENSG00000221968 | FADS3 | protein_coding |
| ENSG00000110400 | NECTIN1 | protein_coding |
| ENSG00000168056 | LTBP3 | protein_coding |
| ENSG00000142949 | PTPRF | protein_coding |
| ENSG00000246582 | AC100861.1 | long_non_coding |
| ENSG00000163814 | CDCP1 | protein_coding |
| ENSG00000274605 | AL355338.1 | long_non_coding |
| ENSG00000124942 | AHNAK | protein_coding |
| ENSG00000186952 | TMEM232 | protein_coding |
| ENSG00000272692 | AC010997.4 | long_non_coding |
| ENSG00000271614 | ATP2B1-AS1 | long_non_coding |
| ENSG00000168528 | SERINC2 | protein_coding |
| ENSG00000257557 | PPP1R12A-AS1 | long_non_coding |
| ENSG00000242110 | AMACR | protein_coding |
| ENSG00000030419 | IKZF2 | protein_coding |
| ENSG00000183386 | FHL3 | protein_coding |
| ENSG00000147251 | DOCK11 | protein_coding |
| ENSG00000261094 | AC007066.2 | long_non_coding |
| ENSG00000167775 | CD320 | protein_coding |
| ENSG00000089163 | SIRT4 | protein_coding |
| ENSG00000181588 | MEX3D | protein_coding |
| ENSG00000142765 | SYTL1 | protein_coding |
| ENSG00000171533 | MAP6 | protein_coding |
| ENSG00000228878 | SEPT7-AS1 | long_non_coding |
| ENSG00000072422 | RHOBTB1 | protein_coding |
| ENSG00000163637 | PRICKLE2 | protein_coding |
| ENSG00000134369 | NAV1 | protein_coding |
| ENSG00000169026 | MFSD7 | protein_coding |
| ENSG00000084444 | FAM234B | protein_coding |
| ENSG00000120899 | PTK2B | protein_coding |
| ENSG00000100968 | NFATC4 | protein_coding |
| ENSG00000245213 | AC105285.1 | long_non_coding |
| ENSG00000176597 | B3GNT5 | protein_coding |
| ENSG00000117245 | KIF17 | protein_coding |
| ENSG00000127990 | SGCE | protein_coding |
| ENSG00000128944 | KNSTRN | protein_coding |
| ENSG00000178718 | RPP25 | protein_coding |
| ENSG00000053918 | KCNQ1 | protein_coding |
| ENSG00000272631 | AC067750.1 | long_non_coding |
| ENSG00000062716 | VMP1 | protein_coding |
| ENSG00000183508 | FAM46C | protein_coding |
| ENSG00000253930 | TNFRSF10A-AS1 | long_non_coding |
| ENSG00000084636 | COL16A1 | protein_coding |
| ENSG00000179218 | CALR | protein_coding |
| ENSG00000116991 | SIPA1L2 | protein_coding |
| ENSG00000259330 | INAFM2 | protein_coding |
| ENSG00000100583 | SAMD15 | protein_coding |
| ENSG00000133640 | LRRIQ1 | protein_coding |
| ENSG00000172667 | ZMAT3 | protein_coding |
| ENSG00000167880 | EVPL | protein_coding |
| ENSG00000260401 | AP002761.4 | long_non_coding |
| ENSG00000087266 | SH3BP2 | protein_coding |
| ENSG00000102554 | KLF5 | protein_coding |
| ENSG00000272944 | AC079834.2 | long_non_coding |
| ENSG00000140450 | ARRDC4 | protein_coding |
| ENSG00000273305 | AC009237.15 | long_non_coding |
| ENSG00000156966 | B3GNT7 | protein_coding |
| ENSG00000187091 | PLCD1 | protein_coding |
| ENSG00000147231 | CXorf57 | protein_coding |
| ENSG00000151948 | GLT1D1 | protein_coding |
| ENSG00000065675 | PRKCQ | protein_coding |
| ENSG00000153885 | KCTD15 | protein_coding |
| ENSG00000162804 | SNED1 | protein_coding |
| ENSG00000151376 | ME3 | protein_coding |
| ENSG00000119326 | CTNNAL1 | protein_coding |
| ENSG00000196923 | PDLIM7 | protein_coding |
| ENSG00000168461 | RAB31 | protein_coding |
| ENSG00000166821 | PEX11A | protein_coding |
| ENSG00000115129 | TP53I3 | protein_coding |
| ENSG00000155760 | FZD7 | protein_coding |
| ENSG00000272398 | CD24 | protein_coding |
| ENSG00000105655 | ISYNA1 | protein_coding |
| ENSG00000188659 | SAXO2 | protein_coding |
| ENSG00000181690 | PLAG1 | protein_coding |
| ENSG00000188643 | S100A16 | protein_coding |
| ENSG00000104723 | TUSC3 | protein_coding |
| ENSG00000109927 | TECTA | protein_coding |
| ENSG00000136478 | TEX2 | protein_coding |
| ENSG00000158079 | PTPDC1 | protein_coding |
| ENSG00000119986 | AVPI1 | protein_coding |
| ENSG00000103064 | SLC7A6 | protein_coding |
| ENSG00000116761 | CTH | protein_coding |
| ENSG00000128294 | TPST2 | protein_coding |
| ENSG00000147912 | FBXO10 | protein_coding |
| ENSG00000127824 | TUBA4A | protein_coding |
| ENSG00000115594 | IL1R1 | protein_coding |
| ENSG00000104324 | CPQ | protein_coding |
| ENSG00000173715 | C11orf80 | protein_coding |
| ENSG00000182168 | UNC5C | protein_coding |
| ENSG00000225471 | BX119917.1 | pseudogene |
| ENSG00000086619 | ERO1B | protein_coding |
| ENSG00000198937 | CCDC167 | protein_coding |
| ENSG00000129422 | MTUS1 | protein_coding |
| ENSG00000109501 | WFS1 | protein_coding |
| ENSG00000267288 | AC138150.2 | long_non_coding |
| ENSG00000176273 | SLC35G1 | protein_coding |
| ENSG00000108379 | WNT3 | protein_coding |
| ENSG00000129474 | AJUBA | protein_coding |
| ENSG00000118804 | STBD1 | protein_coding |
| ENSG00000102048 | ASB9 | protein_coding |
| ENSG00000164045 | CDC25A | protein_coding |
| ENSG00000261572 | AC097639.1 | long_non_coding |
| ENSG00000230487 | PSMG3-AS1 | long_non_coding |
| ENSG00000160606 | TLCD1 | protein_coding |
| ENSG00000166831 | RBPMS2 | protein_coding |
| ENSG00000129116 | PALLD | protein_coding |
| ENSG00000100027 | YPEL1 | protein_coding |
| ENSG00000070081 | NUCB2 | protein_coding |
| ENSG00000166352 | C11orf74 | protein_coding |
| ENSG00000177640 | CASC2 | long_non_coding |
| ENSG00000107821 | KAZALD1 | protein_coding |
| ENSG00000121577 | POPDC2 | protein_coding |
| ENSG00000198720 | ANKRD13B | protein_coding |
| ENSG00000166833 | NAV2 | protein_coding |
| ENSG00000213185 | FAM24B | protein_coding |
| ENSG00000164211 | STARD4 | protein_coding |
| ENSG00000156381 | ANKRD9 | protein_coding |
| ENSG00000213160 | KLHL23 | protein_coding |
| ENSG00000144712 | CAND2 | protein_coding |
| ENSG00000138162 | TACC2 | protein_coding |
| ENSG00000224848 | AL589843.1 | long_non_coding |
| ENSG00000196586 | MYO6 | protein_coding |
| ENSG00000167470 | MIDN | protein_coding |
| ENSG00000260063 | AL512408.1 | long_non_coding |
| ENSG00000148426 | PROSER2 | protein_coding |
| ENSG00000177426 | TGIF1 | protein_coding |
| ENSG00000072310 | SREBF1 | protein_coding |
| ENSG00000141524 | TMC6 | protein_coding |
| ENSG00000247400 | DNAJC3-AS1 | long_non_coding |
| ENSG00000166669 | ATF7IP2 | protein_coding |
| ENSG00000119899 | SLC17A5 | protein_coding |
| ENSG00000179168 | GGN | protein_coding |
| ENSG00000117298 | ECE1 | protein_coding |
| ENSG00000100600 | LGMN | protein_coding |
| ENSG00000139537 | CCDC65 | protein_coding |
| ENSG00000167037 | SGSM1 | protein_coding |
| ENSG00000100116 | GCAT | protein_coding |
| ENSG00000146242 | TPBG | protein_coding |
| ENSG00000273311 | DGCR11 | long_non_coding |
| ENSG00000258701 | LINC00638 | long_non_coding |
| ENSG00000171219 | CDC42BPG | protein_coding |
| ENSG00000167191 | GPRC5B | protein_coding |
| ENSG00000115993 | TRAK2 | protein_coding |
| ENSG00000152104 | PTPN14 | protein_coding |
| ENSG00000078804 | TP53INP2 | protein_coding |
| ENSG00000159263 | SIM2 | protein_coding |
| ENSG00000162174 | ASRGL1 | protein_coding |
| ENSG00000137831 | UACA | protein_coding |
| ENSG00000007384 | RHBDF1 | protein_coding |
| ENSG00000276073 | AL034549.2 | long_non_coding |
| ENSG00000171132 | PRKCE | protein_coding |
| ENSG00000152642 | GPD1L | protein_coding |
| ENSG00000151150 | ANK3 | protein_coding |
| ENSG00000158710 | TAGLN2 | protein_coding |
| ENSG00000223768 | LINC00205 | long_non_coding |
| ENSG00000203288 | TDRKH-AS1 | long_non_coding |
| ENSG00000147065 | MSN | protein_coding |
| ENSG00000227855 | DPY19L2P3 | pseudogene |
| ENSG00000266028 | SRGAP2 | protein_coding |
| ENSG00000267534 | S1PR2 | protein_coding |
| ENSG00000126458 | RRAS | protein_coding |
| ENSG00000108448 | TRIM16L | protein_coding |
| ENSG00000116729 | WLS | protein_coding |
| ENSG00000169116 | PARM1 | protein_coding |
| ENSG00000149054 | ZNF215 | protein_coding |
| ENSG00000148158 | SNX30 | protein_coding |
| ENSG00000063180 | CA11 | protein_coding |
| ENSG00000102349 | KLF8 | protein_coding |
| ENSG00000170469 | SPATA24 | protein_coding |
| ENSG00000115170 | ACVR1 | protein_coding |
| ENSG00000132436 | FIGNL1 | protein_coding |
| ENSG00000069974 | RAB27A | protein_coding |
| ENSG00000280195 | AC245140.2 | long_non_coding |
| ENSG00000173320 | STOX2 | protein_coding |
| ENSG00000143067 | ZNF697 | protein_coding |
| ENSG00000179240 | AP002360.1 | protein_coding |
| ENSG00000235280 | MCF2L-AS1 | long_non_coding |
| ENSG00000221926 | TRIM16 | protein_coding |
| ENSG00000159259 | CHAF1B | protein_coding |
| ENSG00000177694 | NAALADL2 | protein_coding |
| ENSG00000158716 | DUSP23 | protein_coding |
| ENSG00000178919 | FOXE1 | protein_coding |
| ENSG00000119318 | RAD23B | protein_coding |
| ENSG00000196781 | TLE1 | protein_coding |
| ENSG00000132481 | TRIM47 | protein_coding |
| ENSG00000166401 | SERPINB8 | protein_coding |
| ENSG00000161013 | MGAT4B | protein_coding |
| ENSG00000234456 | MAGI2-AS3 | long_non_coding |
| ENSG00000244607 | CCDC13 | protein_coding |
| ENSG00000205309 | NT5M | protein_coding |
| ENSG00000274220 | AC009163.7 | long_non_coding |
| ENSG00000148225 | WDR31 | protein_coding |
| ENSG00000134109 | EDEM1 | protein_coding |
| ENSG00000152990 | ADGRA3 | protein_coding |
| ENSG00000112667 | DNPH1 | protein_coding |
| ENSG00000159840 | ZYX | protein_coding |
| ENSG00000110002 | VWA5A | protein_coding |
| ENSG00000163013 | FBXO41 | protein_coding |
| ENSG00000225265 | TAF1A-AS1 | long_non_coding |
| ENSG00000260912 | AL158206.1 | long_non_coding |
| ENSG00000180769 | WDFY3-AS2 | long_non_coding |
