# Supplementary material for: Identification of LINC02454-related key pathways and genes in papillary thyroid cancer by weighted gene coexpression network analysis (WGCNA)
Source: Thyroid Res. 2024 Sep 2;17:17. doi: 10.1186/s13044-024-00205-8 (PMC11367880; doi:10.1186/s13044-024-00205-8)
Supplement: Supplementary file 4 — Supplementary Material 4. [file 13044_2024_205_MOESM4_ESM.docx]

Supplementary Table 2 The genes co-expressed with LINC02454 in the turquoise module

| fromNode | toNode | weight |
| --- | --- | --- |
| PDLIM4 | LINC02454 | 0.169837621 |
| PTPRE | LINC02454 | 0.169805324 |
| SERPINA1 | LINC02454 | 0.162519765 |
| LINC02454 | SDC4 | 0.158840475 |
| SLC22A31 | LINC02454 | 0.150098807 |
| SYT12 | LINC02454 | 0.149535788 |
| PROS1 | LINC02454 | 0.14849429 |
| TIMP1 | LINC02454 | 0.147950884 |
| LINC02454 | LGALS3 | 0.145459919 |
| TACSTD2 | LINC02454 | 0.14224866 |
| TMPRSS4 | LINC02454 | 0.139066924 |
| KCNN4 | LINC02454 | 0.136326497 |
| FN1 | LINC02454 | 0.134600862 |
| KCNQ3 | LINC02454 | 0.13455395 |
| LINC02454 | ANXA1 | 0.132943513 |
| RUNX2 | LINC02454 | 0.132943108 |
| LINC02454 | LIPH | 0.132871635 |
| LINC02471 | LINC02454 | 0.132288757 |
| LINC02454 | TMC6 | 0.132042185 |
| NOD1 | LINC02454 | 0.131879402 |
| GJB3 | LINC02454 | 0.130825444 |
| AC025419.1 | LINC02454 | 0.130154898 |
| LAMB3 | LINC02454 | 0.129642521 |
| COL8A2 | LINC02454 | 0.128488512 |
| GABRB2 | LINC02454 | 0.128237542 |
| PDE5A | LINC02454 | 0.12754611 |
| CAMK2N1 | LINC02454 | 0.127483396 |
| NECTIN4 | LINC02454 | 0.127385318 |
| LINC02454 | MTMR11 | 0.127335697 |
| LINC02454 | PLCD3 | 0.12725889 |
| LINC02454 | TNRC6C-AS1 | 0.126139045 |
| LINC02454 | STK32A-AS1 | 0.125892362 |
| RASGRF1 | LINC02454 | 0.125586115 |
| ZCCHC12 | LINC02454 | 0.125452739 |
| LINC02454 | GALE | 0.125383831 |
| RXRG | LINC02454 | 0.12428448 |
| CLDN10 | LINC02454 | 0.123515249 |
| PLEKHN1 | LINC02454 | 0.123277561 |
| TMPRSS6 | LINC02454 | 0.123132365 |
| ICAM1 | LINC02454 | 0.12287504 |
| CYP2S1 | LINC02454 | 0.122631464 |
| AP002358.1 | LINC02454 | 0.121691877 |
| SLC34A2 | LINC02454 | 0.121641812 |
| LINC02454 | STK32A | 0.121236025 |
| LINC02454 | GRB7 | 0.121005003 |
| LINC02454 | B3GNT7 | 0.120947837 |
| LRRK2 | LINC02454 | 0.12044747 |
| ITGA3 | LINC02454 | 0.119870108 |
| RUNX1 | LINC02454 | 0.119620705 |
| LINC02454 | GLT1D1 | 0.11950324 |
| MET | LINC02454 | 0.119238968 |
| SLC25A47P1 | LINC02454 | 0.118941594 |
| LINC02454 | PLAG1 | 0.118366328 |
| LINC02454 | DIRAS3 | 0.118251535 |
| TMEM92 | LINC02454 | 0.118190568 |
| LINC02454 | METTL7B | 0.117838743 |
| EPHB3 | LINC02454 | 0.117605471 |
| AL157714.2 | LINC02454 | 0.116958707 |
| LRP4 | LINC02454 | 0.116640887 |
| DCSTAMP | LINC02454 | 0.116373813 |
| HMGA2 | LINC02454 | 0.116317017 |
| LINC02555 | LINC02454 | 0.116205952 |
| LINC02454 | GOLT1A | 0.116020023 |
| LINC02454 | HEY2 | 0.115315072 |
| PRR15 | LINC02454 | 0.114384008 |
| KRT19 | LINC02454 | 0.114159315 |
| LINC02454 | CDH3 | 0.113814059 |
| LINC02454 | RPSAP52 | 0.113754558 |
| IL1RAP | LINC02454 | 0.113140206 |
| NGEF | LINC02454 | 0.112984863 |
| KRT15 | LINC02454 | 0.112888931 |
| RTL4 | LINC02454 | 0.112537213 |
| LINC02454 | MVP | 0.112212738 |
| TENM1 | LINC02454 | 0.111856401 |
| SLC27A6 | LINC02454 | 0.111533542 |
| LINC02454 | ACACB | 0.111520016 |
| CITED1 | LINC02454 | 0.111365199 |
| LINC02454 | ABCC3 | 0.11035253 |
| AL365259.1 | LINC02454 | 0.11019811 |
| LINC02454 | TGFA | 0.110144125 |
| CATSPER1 | LINC02454 | 0.109904255 |
| AC079630.1 | LINC02454 | 0.109642511 |
| MAMLD1 | LINC02454 | 0.109376456 |
| REN | LINC02454 | 0.109186893 |
| SSTR3 | LINC02454 | 0.108263595 |
| AC007255.1 | LINC02454 | 0.108185087 |
| KATNAL2 | LINC02454 | 0.108046127 |
| IGFBP6 | LINC02454 | 0.107938938 |
| QPCT | LINC02454 | 0.107513677 |
| LINC02454 | BID | 0.107398637 |
| HPCAL4 | LINC02454 | 0.107239943 |
| B3GNT3 | LINC02454 | 0.107108324 |
| CTSH | LINC02454 | 0.107050942 |
| MACC1 | LINC02454 | 0.107026406 |
| LINC02454 | AC120036.5 | 0.106738099 |
| LINC02454 | GALNT7 | 0.106479442 |
| SYNDIG1L | LINC02454 | 0.106088573 |
| SLC4A4 | LINC02454 | 0.10570884 |
| DTX4 | LINC02454 | 0.105515155 |
| DUSP5 | LINC02454 | 0.105200946 |
| AC005479.3 | LINC02454 | 0.104751225 |
| KLHDC8A | LINC02454 | 0.104739302 |
| LINC02454 | ADAMTS9-AS2 | 0.104684021 |
| CREB5 | LINC02454 | 0.104663698 |
| LINC02454 | MPZL2 | 0.104285522 |
| LINC02454 | TUSC3 | 0.103719583 |
| AC254633.1 | LINC02454 | 0.103588402 |
| SYTL5 | LINC02454 | 0.103255276 |
| LINC02454 | RAB27A | 0.103158657 |
| FBN3 | LINC02454 | 0.102911711 |
| LINC02454 | LPAR5 | 0.102772328 |
| LINC02454 | TNFRSF12A | 0.102770538 |
| LINC02454 | SPAG5 | 0.102729859 |
| EVA1A | LINC02454 | 0.10260342 |
| IL17RD | LINC02454 | 0.102523848 |
| LINC02454 | AC105285.1 | 0.102121128 |
| SYN1 | LINC02454 | 0.10195724 |
| LINC02454 | MXRA8 | 0.101896275 |
| SNX22 | LINC02454 | 0.101853992 |
| ADGRB3 | LINC02454 | 0.101656234 |
| RSPO4 | LINC02454 | 0.101457918 |
| SCG5 | LINC02454 | 0.1013569 |
| LINC02454 | C11orf80 | 0.101211915 |
| RDH5 | LINC02454 | 0.101105422 |
| LINC02454 | AC126773.2 | 0.101018435 |
| ICAM5 | LINC02454 | 0.101003489 |
| TIMM8AP1 | LINC02454 | 0.100696934 |
| LINC02454 | CLDN1 | 0.100685771 |
| EGFEM1P | LINC02454 | 0.100472296 |
| TBC1D2 | LINC02454 | 0.100298338 |
| LINC02454 | CBARP | 0.100252668 |
