# Supplementary material for: Identification of LINC02454-related key pathways and genes in papillary thyroid cancer by weighted gene coexpression network analysis (WGCNA)
Source: Thyroid Res. 2024 Sep 2;17:17. doi: 10.1186/s13044-024-00205-8 (PMC11367880; doi:10.1186/s13044-024-00205-8)
Supplement: Supplementary file 5 — Supplementary Material 5. [file 13044_2024_205_MOESM5_ESM.docx]

Supplementary Table 3 Top 40 LINC02454 co-expressed

genes in the network by MCC method

| Gene name | Score |
| --- | --- |
| PTPRE | 505 |
| SYT12 | 463 |
| PROS1 | 394 |
| CYP2S1 | 391 |
| TMPRSS4 | 372 |
| FN1 | 361 |
| SDC4 | 350 |
| TACSTD2 | 337 |
| EVA1A | 334 |
| PDLIM4 | 327 |
| KCNQ3 | 325 |
| RUNX1 | 308 |
| ST6GALNAC5 | 308 |
| SLC25A47P1 | 307 |
| TMPRSS6 | 307 |
| COL8A2 | 298 |
| SLC34A2 | 297 |
| CLDN10 | 287 |
| PLCD3 | 272 |
| TMEM92 | 270 |
| SERPINA1 | 265 |
| TIMP1 | 259 |
| ANXA1 | 250 |
| CREB5 | 240 |
| B3GNT3 | 240 |
| NOD1 | 227 |
| STAC | 225 |
| NECTIN4 | 223 |
| ITGA3 | 222 |
| CATSPER1 | 221 |
| GABRB2 | 220 |
| LGALS3 | 218 |
| RUNX2 | 216 |
| IL1RAP | 211 |
| KCNN4 | 210 |
| PLEKHN1 | 204 |
| MVP | 200 |
| SLC22A31 | 194 |
| LAMB3 | 192 |
| EPHB3 | 188 |
