# Supplementary material for: Identification of LINC02454-related key pathways and genes in papillary thyroid cancer by weighted gene coexpression network analysis (WGCNA)
Source: Thyroid Res. 2024 Sep 2;17:17. doi: 10.1186/s13044-024-00205-8 (PMC11367880; doi:10.1186/s13044-024-00205-8)
Supplement: Supplementary file 6 — Supplementary Material 6. [file 13044_2024_205_MOESM6_ESM.docx]

Supplementary Table 4 Gene clusters involved in KEGG pathways enrichment

| Source | Description | Gene name |
| --- | --- | --- |
| hsa04512 | ECM-receptor interaction | FN1,TNC,COL9A3,ITGA2,LAMC3,LAMB3,ITGB6,  ITGB4,ITGB8,LAMA1,FREM2,ITGA3,SDC4,COL4A5,  LAMC2,ITGA9,ITGA7 |
| hsa04010 | MAPK signaling pathway | CACNG4,NGFR,RASGRF1,AREG,KIT,PLA2G4F,DUSP5,  BDNF,PGF,CACNA2D3,IL1RAP,CACNB4,PRKCA,MET,  ERBB3,TGFBR1,FGF2,CACNB2,VEGFA,GADD45A,FAS,  MAP3K5,MAPT,MAP2K6,TGFA,DUSP6,RAC3,RPS6KA6,  FLNA,DUSP8,EGFR,CACNB1,PRKACB,CDC25B,DUSP4,  EPHA2,MAPK10,IL1R1,RRAS |
| hsa05205 | Proteoglycans in cancer | FN1,WNT10A,WNT4,ITGA2,ITPR1,ESR1,VAV3,HBEGF,  CAV1,HPSE,TIAM1,PRKCA,MET,ERBB3,FGF2,SDC4,  VEGFA,CAV2,FAS,FZD8,FLNA,WNT5A,EGFR,PRKACB,  FZD7,WNT3,ANK3,MSN,RRAS |
| hsa04931 | Insulin resistance | SLC27A6,PPARGC1A,PYGM,CREB5,SLC2A4,MLXIPL,IRS1,  PPP1R3C,TBC1D4,IRS2,RPS6KA6,ACACB,SLC27A2,MAPK10,  PTPRF,PRKCQ,SREBF1,PRKCE |
| hsa04918 | Thyroid hormone synthesis | ADCY8,TPO,SLC26A4,IYD,LRP2,ITPR1,CREB5,TG,ADCY1,PRKCA,  ,GPX3,HSPA5,PRKACB,GNAS |
| hsa05222 | Small cell lung cancer | BIRC7,FN1,ITGA2,PTGS2,LAMC3,LAMB3,RXRG,LAMA1,  CDKN2B,ITGA3,BCL2,RARB,GADD45A,COL4A5,LAMC2,  ,BCL2L1 |
| hsa00230 | Purine metabolism | ADCY8,PDE11A,ENTPD2,PNP,PDE10A,NT5E,PDE4C,ENTPD8,  PDE8B,ADCY1,PDE5A,ENTPD3,GUCY2D,PDE4B,PDE4D,PDE9A,  AK4,AK7,ENTPD1,NT5M |
| hsa04215 | Apoptosis -multiple species | BIRC7,NGFR,PMAIP1,BCL2,BBC3,BCL2L1,BID,MAPK10 |
| hsa05217 | Basal cell carcinoma | WNT10A,WNT4,TCF7L1,BMP4,AXIN2,GADD45A,TCF7,FZD8,  WNT5A,GLI3,FZD7,WNT3 |
| hsa04750 | Inflammatory mediator regulation of TRP channels | ADCY8,PLA2G4F,ITPR1,HRH1,IL1RAP,ADCY1,PRKCA,MAP2K6,  P2RY2,PRKACB,GNAS,F2RL1,MAPK10,PRKCQ,IL1R1,PRKCE |
